# Extracellular Vesicles: Novel Opportunities to Understand and Detect Neoplastic Diseases

**DOI:** 10.1177/0300985821999328

**Published:** 2021-04-05

**Authors:** Laura Bongiovanni, Anneloes Andriessen, Marca H. M. Wauben, Esther N. M. Nolte-’t Hoen, Alain de Bruin

**Affiliations:** 1 90051 Utrecht University, Utrecht, the Netherlands; 2 University Medical Center Groningen, University of Groningen, Groningen, the Netherlands; * Present address: Faculty of Veterinary Medicine, University of Teramo, Teramo, Italy.

**Keywords:** biomarker, cancer, exosomes, extracellular vesicles, microvesicles pathogenesis, pathology

## Abstract

With a size range from 30 to 1000 nm, extracellular vesicles (EVs) are one of the smallest cell components able to transport biologically active molecules. They mediate intercellular communications and play a fundamental role in the maintenance of tissue homeostasis and pathogenesis in several types of diseases. In particular, EVs actively contribute to cancer initiation and progression, and there is emerging understanding of their role in creation of the metastatic niche. This fact underlies the recent exponential growth in EV research, which has improved our understanding of their specific roles in disease and their potential applications in diagnosis and therapy. EVs and their biomolecular cargo reflect the state of the diseased donor cells, and can be detected in body fluids and exploited as biomarkers in cancer and other diseases. Relatively few studies have been published on EVs in the veterinary field. This review provides an overview of the features and biology of EVs as well as recent developments in EV research including techniques for isolation and analysis, and will address the way in which the EVs released by diseased tissues can be studied and exploited in the field of veterinary pathology. Uniquely, this review emphasizes the important contribution that pathologists can make to the field of EV research: pathologists can help EV scientists in studying and confirming the role of EVs and their molecular cargo in diseased tissues and as biomarkers in liquid biopsies.

Extracellular vesicles (EVs) play an important roles in the maintenance of tissue homeostasis and pathogenesis. Due to their small size, we are unable to see EVs by light microscopy, and some of them can only be visualized by electron microscopy, eventually with the use of immunogold labeling using specific EV markers. Several techniques have been developed for the collection and analysis of EVs from body fluids such as blood, but it remains extremely difficult to isolate them directly from tissues. EVs are effectively messages sent by cells that contain specific “words” (bioactive molecules) and are used by cells to communicate with other cells. Thus, the content of EVs is very specific and makes them a highly attractive research topic. A growing body of research aims to better elucidate the roles of EVs in tissue development, maintenance, and function, as well as in pathogenesis. Noteworthy, EV messages can have a local or distant effect: They can act as paracrine agents when they are released into the extracellular space or as endocrine agents when they are released in the circulation and thereby affect distant organs and cells. The molecular content of EVs in the blood or in other body fluids can provide information about their tissue of origin, allowing them to be used as biomarkers. As EVs can target specific tissues and be taken up by specific cells, EVs can be exploited to convey and deliver therapeutic molecules.

EV research in veterinary medicine is still at an early stage and the literature is limited but enough to show the potential of EV application in different fields of animal research and types of disease: the published topics include canine and feline cancers and kidney diseases, bovine mammary and metabolic disorders, and equine and bovine infectious diseases. However, the majority of these studies are descriptive, for example, isolating and characterizing EV size, morphology, antigens, or molecular cargo. However, functional studies with rigorous experimental validation are necessary to prove the role of EVs in pathogenesis, such as studies where EVs are transferred in in vitro or in vivo models and consequent changes in gene expression, protein levels, or phenotypes are documented.^80^

Referring both to human and veterinary research works, the present review is intended to briefly explain EVs’ biology and their roles in tissue homeostasis, provide deeper insight in their role in neoplasia and discuss potential applications of EVs as biomarkers in different types of diseases. The current techniques to isolate and analyze EVs will be also described, with their main advantages and disadvantages. A final section describes on how pathologists contribute to EV research, providing new perspectives and ideas for researchers and pathologists interested in working in this filed.

## EV Classification and Biology

**Figure 1.**
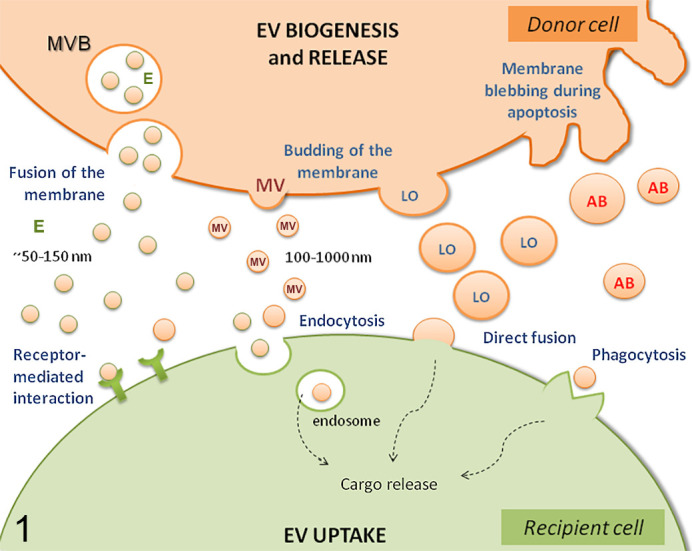
Mechanisms of extracellular vesicle (EV) biogenesis, release, and uptake. Different types of EVs are produced and released in different ways by donor cells: by formation of multivesicular bodies and fusion to the membrane (exosome), or by direct blebbing of the membrane (microvesicles, large oncosome, apoptotic bodies). Once in the intercellular space, EVs can be uptaken by target cells by receptor-mediated recognition, endocytosis, phagocytosis, or direct fusion with the plasma membrane. MVB, multivesicular body; E, exosome; MV, microvesicle; LO, large oncosome; AB, apoptotic body.

All EVs are naturally released by cells, are surrounded by a lipid bilayer, and cannot replicate. Based on this definition provided by the Minimal Information for Studies of Extracellular Vesicles 2018 (MISEV2018),^191^ EVs are part of the complete secretome of the cell and there are no specific markers to distinguish EV subtypes and their subcellular origin. However, differences exist that enable categorization of EVs into distinct subclasses. There are 2 main classes of EVs—exosomes and microvesicles—that mainly differ in their mode of biogenesis rather than their size (Table 1, Fig. 1). *Exosomes* are small EVs (∼50–150 nm diameter) that arise in the endosomal system. The endosomal system consists of highly dynamic membrane compartments that actively interact to regulate the uptake of molecules or ligands, their recycling to the cell surface, and their degradation.^73^ Endosomes provide an intracellular environment where molecules can be sorted prior to determining their fate. Inward budding of the endosomal limiting membrane leads to the formation of multivesicular bodies that direct molecules to lysosomes for degradation or to the plasma membrane for release into the extracellular space. Intraluminal vesicles arise in multivesicular bodies through budding mediated by the endosomal sorting complex required for transport complexes.^77^ These vesicles are released as exosomes into the extracellular environment upon fusion of multivesicular bodies with the plasma membrane.^33^ *Microvesicles*, also referred to as *ectosomes*, are larger EVs of 100 to 1000 nm diameter that are released into the extracellular space by direct budding from the plasma membrane. They also include microvesicles released from specific cell types, such as from apoptotic cells or tumor cells. In these circumstances they are typically referred to as *apoptotic bodies* and *large oncosomes*, respectively. The cargo sorting and outward plasma membrane budding resulting in microvesicle release is mediated by small GTPases as well as components of the endosomal sorting complex required for transport machinery.^31,139,140,203^ Since there can be some overlap in the size of exosomes and microvesicles, classification is primarily based on the mode of biogenesis.

**Table 1.** Classification of EV Based on Their Size, Density, and Mode of Biogenesis.^a^

EV type	Diameter (nm)	Density (g/mL)	Cellular origin	Origin
Exosomes	30–150^b^	1.13–1.19	Most cell types	MVB
	<100^c^	1.10–1.18^c^		
Microvesicles	200–1000^b^	1.04–1.07^c^	Most cell types	PM-shed vesicle
	100–1000^c^			
Apoptotic bodies	1000 to >5000	1.16–1.28	All cell types	PM-shed vesicle
	500–4000^c^			
Large oncosomes^d^	1000–10 000^d^	1.10–1.15^e^	Tumor cells^d^	PM-shed vesicle^d^

Abbreviations: MVB, multivesicular bodies; PM, plasma membrane.

^a^ Adapted from van der Pol E, Böoing AN, Sturk A, et al. Classification, functions, and clinical relevance of extracellular vesicles. *Pharmacol Rev*. **64**(3):676–705.

^b^ Whiteside et al.^205^

^c^ Samanta et al.^170^

^d^ Ciardiello et al.^33^

^e^ Minciacchi et al.^127^

Based on MISEV2018, *microparticles* fall under the definition of “EV,”^191^ rendering the 2 terms at least partially overlapping, and no clear definition of microparticles is available. However, several articles have been published in veterinary medicine on the role of microparticles, especially platelet-derived microparticles, in coagulation and cardiovascular diseases.^94,96^

After release by donor cells into the extracellular space, EVs reach their target cells. Many EVs are taken up by the recipient cells and degraded by their lysosomal system; in others, the EV contents induce phenotypic changes in the recipient cell. EVs can transmit information both at the recipient cell surface and after internalization. Uptake of EVs requires that EVs bind to specific receptors present on the surface of target cells.^146^ However, it is not known if binding of particular EV subtypes to recipient cells is target-specific or nonspecific and stochastic; it is likely that both mechanisms occur.^118^ The various mechanisms by which EVs are internalized into the recipient cell seem to be more dependent on the recipient cell type than on the EVs themselves. EVs can directly fuse to the plasma membrane of the recipient cells and then release their content into the cytoplasm. Alternatively, EVs can be internalized by phagocytosis or endocytosis. Endocytosis can be clathrin-dependent or clathrin-independent (lipid raft-mediated). The latter can require the presence of caveolins, which are proteins involved in the creation of small cave-like invaginations in the plasma membrane.^61^ Endocytosis can result in EV degradation in the lysosome or release of the EV cargo into the cytoplasm of the recipient cells by back-fusion with the endosomal membrane.^150^ However, investigation of this final step in EV uptake, namely, the delivery of EV contents into the recipient cell via EV degradation or re-secretion, is crucial to understand the functional consequences of EV-mediated transfer of bioactive molecules.^118^

## Physiological Roles of EVs

**Figure 2.**
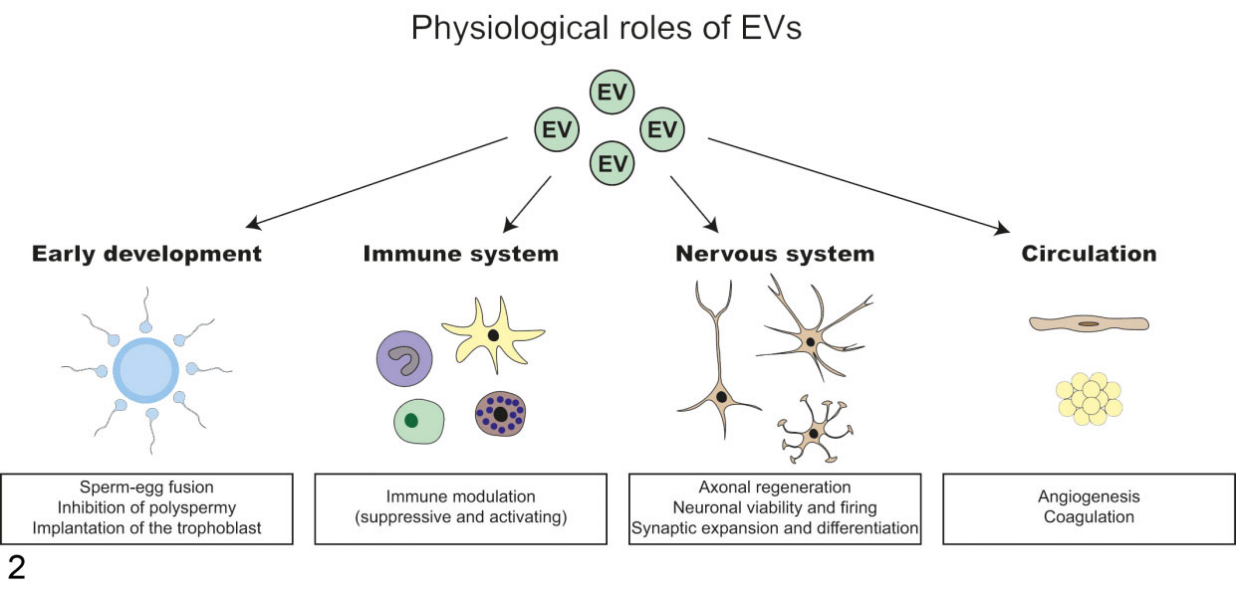
Roles of extracellular vesicles (EVs) in physiological processes. A variety of cell types in the body communicate via EVs. EV-mediated transfer of bioactive cargo influences processes in early development, the immune system, nervous system, and circulatory system.

Research over the past decade has demonstrated that EVs are not only generated by cells during disease but are also secreted by healthy cells where they mediate intercellular communication in a number of physiological processes. Here follows a brief overview of the physiological processes in which EVs play an important role, ranging from embryonic development to maintenance of tissue homeostasis (Fig. 2).

### EVs in Conception and Early Development

The presence of EVs has been demonstrated in the seminal fluid of multiple species including humans. The proteins contained within these EV (eg, adhesion molecules, enzymes of the polyol pathway) play a role in sperm maturation and fertilization.^62,68,192^ Specifically, CD9-carrying EVs promote sperm-egg fusion.^129^ After fertilization, EV shedding is utilized to remove the sperm receptor from the plasma membrane of the oocyte in order to prevent polyspermy.^14^ Interestingly, embryonic stem cells (ESCs) residing in the inner cell mass of the blastocyst also release EVs that they use to communicate with their environment. ESCs release EVs that are transferred to trophoblasts stimulating their migration and invasive properties.^43^ This suggests that the embryo itself contributes to the highly coordinated process of embryo implantation through EV-mediated communication, a finding that was confirmed in in vivo experiments where injection of blastocysts with ESC-derived EVs enhanced the implantation rate in mice.

### EVs in Immune Regulation

EV-mediated communication is involved in both the adaptive and innate immune responses. Antigen-presenting cells including B lymphocytes generate EVs carrying peptide-MHC complexes that activate T cell lines, primed CD4+ T cells, and T lymphocytes in vivo.^2,138,146,164,219^ EVs derived from antigen-presenting cells also carry RNA cargo that influences immune cell behavior. For example, EV-mediated transfer of miRNAs between dendritic cells represses target mRNA expression in the acceptor dendritic cells.^132^ T lymphocytes also use EVs to transfer miRNAs to antigen-presenting cells at the immune synapse and alter their gene expression profiles.^128^ Regulatory T cells secrete EVs containing miRNAs that are taken up by T helper 1 cells and suppress their inflammatory responses.^149^ Tumor-derived EV-enclosed miRNAs bind to Toll-like receptors on macrophages, resulting in activation of the macrophage immune response.^52^ Contrary to this, EV-mediated miRNA transfer from mesenchymal stem cells to macrophages suppresses Toll-like receptor activation.^159^ Overall, either through antigen presentation or by content transfer, EVs have an important role in modulating immune responses (reviewed in Robbins and Morelli^167^).

### EVs in the Nervous System

Neurons and glia cells use EVs to mediate intercellular communication. In vitro cultured neurons release EVs on stimulation of glutamatergic synaptic activity and depolarization.^56,100^ Stimulated neuron-derived EVs were selectively taken up by other neurons and not by glial cells, suggesting a mechanism of interneuronal communication.^30^ The cargo of neuron-derived EVs is functionally active and capable of inducing phenotypic changes in recipient cells. For example, the uptake of EV-enclosed miR-124a by astrocytes induced an upregulation in their expression of the astroglial glutamate transporter GLT1.^136^ EVs released by oligodendrocytes contain myelin proteins and oxidative stress-protective proteins and are taken up by neurons resulting in altered neuronal firing rates and gene expression profiles, although underlying mechanisms remain to be further defined.^63,64,99^ Furthermore, oligodendrocyte-derived EVs regulate oligodendrocyte physiology by inhibiting their differentiation and myelin formation.^11^ Schwann cells release EVs that are taken up by axons and enhance their regenerative capacity after sciatic nerve injury in vivo.^111^ It has been suggested that EVs released by microglia regulate neuronal excitability by inhibiting the synthesis of sphingolipid ceramides and sphingosine.^8^ Sphingosine stimulates exocytosis of vesicles into the synaptic space.^42^ In vitro cultured astrocytes have also been shown to release EVs.^15,45^ The function of these astrocyte-shed EVs remains unclear although they are suggested to play a regulatory role in the immunological response to inflammatory brain lesions.^45^ Collectively, these studies demonstrate that neurons and a variety of glial cells release EVs that modulate neuronal excitability, repair mechanisms, and offer protection against cellular stress.

### EVs in the Circulation

Tissue factor is present on the membranes of vesicles in the blood of healthy human subjects.^67^ This suggests a thrombogenic role for EVs because tissue factor activates the coagulation cascade; however, the majority of circulating tissue factor is still thought to be present in the noncoagulant form.^215^ Human wound blood, on the other hand, has been shown to contain EVs exposing highly procoagulant tissue factor, further supporting a role for EVs in hemostasis.^16,144^

EVs have stimulatory and inhibitory effects on the formation and expansion of new blood vessels. EVs released by endothelial cells carry matrix metalloproteinases that enhance matrix degradation and promote angiogenesis.^190^ Platelet-derived EVs promote endothelial cell proliferation, survival, migration, and vessel formation.^23,95^ In contrast, lymphocyte-derived EVs suppress angiogenesis by disrupting the VEGF signaling pathway and augmenting oxidative stress.^137^

These findings can be potentially applied to the clinical setting. As an example, stem cell–derived EVs and their bioactive cargo have tissue regeneration abilities that may open novel therapeutic avenues for the repair and regeneration of damaged tissues.^114^

## EVs in Pathogenesis: Key Mediators of Intercellular Crosstalk

The secretion of altered EVs likely contributes directly to the pathogenesis of various neoplastic, infectious, degenerative, and immune-mediated diseases. The analysis of EVs and their biologically active cargo may help identify disease mechanisms and form a basis for the development of novel therapeutic approaches.

**Figure 3.**
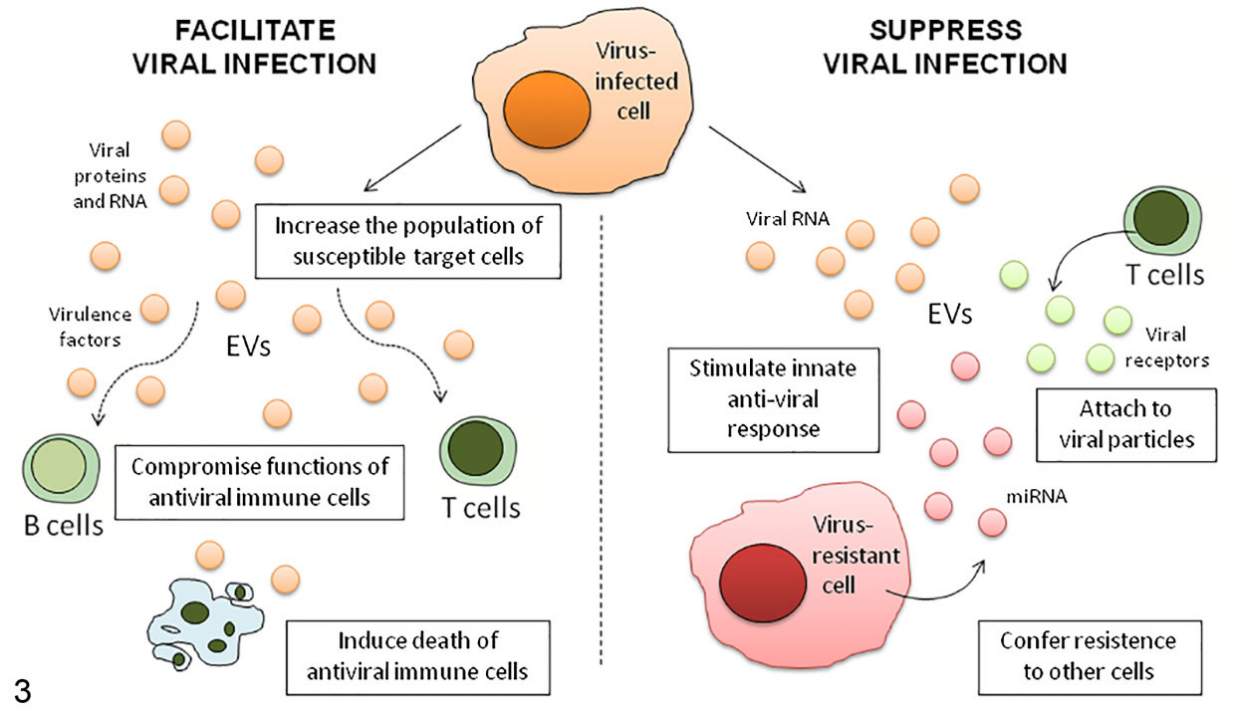
Roles of extracellular vesicles (EVs) during retroviral infection. EVs produced and released by infected cells can both facilitate and suppress viral infection by different mechanisms. EVs can carry viral proteins, receptors, and RNAs. EVs released by inflammatory cells that have been activated by viral infection may also play a key role in the pathogenesis of viral diseases.

Even if this part of the review will be focused on the role of EVs in neoplastic diseases, there are several published works in veterinary medicine investigating the role and potential applications of EVs in infectious and degenerative diseases. Viral and bacterial pathogens change EV content and functions in affected cells to promote their own replication, survival, and pathologic effects. During viral infection, for example, such as retroviral infection (Fig. 3 **)**, a fundamental contribution of EVs has been discovered, strongly linking the fields of EV biology and virology.^72^ Preliminary data on the potential role of EVs in the pathogenesis and virus transmission of viral diseases of animals have been published recently. These include analyses of serum EVs from pigs infected with African swine fever virus^116^ and porcine reproductive and respiratory syndrome virus,^131^ milk EVs from cows infected by bovine leukemia virus,^210^ and EVs from semen of equine stallions with long-term persistent infection by equine arteritis virus.^24^ The small number of studies that have investigated EVs in infectious disease in domestic animals have offered a glimpse of their importance to understand host-pathogen interactions. Furthermore, while research on the role of EVs in degenerative and immune-mediated diseases is just beginning, the numerous spontaneous animal models of these disorders can offer a good research setting to further investigate EVs role and potential applications.

Histological observations of diseased tissues such as the distribution of altered cells, the expression patterns of specific molecules, and how subpopulations of diseased cells are distributed and interconnected to each other can guide pathologists in generating new hypotheses on key pathogenetic mechanisms of intercellular crosstalk mediated by EVs and their cargo. Based on these observations and presumptions, in vitro models can be developed to allow the identification of active molecules contained in the EVs that are released by donor cells and that activate specific cellular pathways when they are taken up by the recipient cells. Subsequently, the functional role of the EV cargo should be confirmed in vivo, through the use of animal models or by analyzing tissue samples obtained from patients. The latter is a key step, but to date still represents a major challenge in EV research. This stepwise approach based on the use of multiple methods and techniques represents an example of how pathologists can contribute to the field.

The body of work performed on the role of EVs in cancer is summarized in the following paragraphs and illustrated by examples from the human and veterinary research fields.

## EVs in the Pathogenesis of Neoplastic Diseases

**Figure 4.**
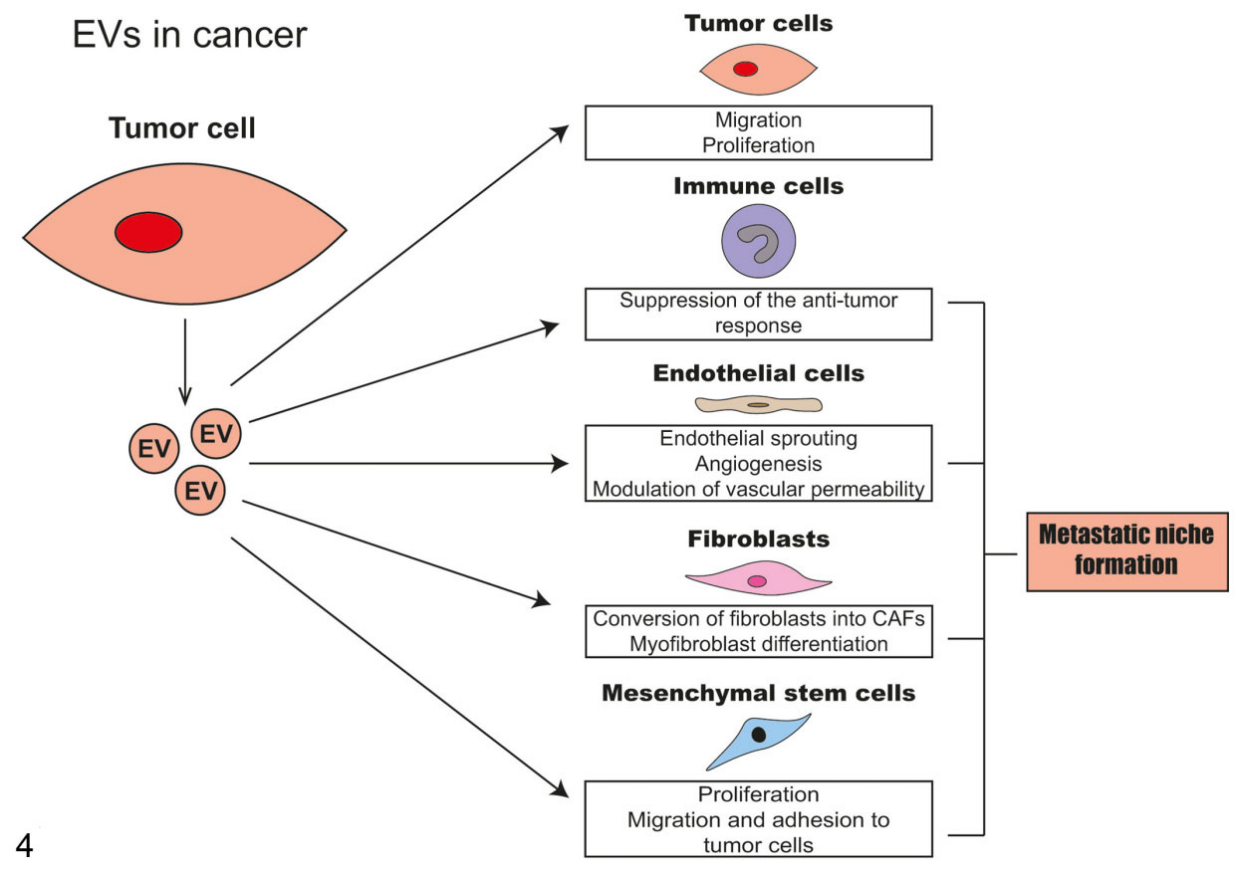
Main roles of cancer-derived extracellular vesicles (EVs) in tumor pathogenesis. Tumor-derived EVs alter the behavior of cancer cells, thereby facilitating cancer progression. Tumor-derived EVs induce alterations in immune cells, endothelial cells, fibroblasts, and mesenchymal stem cells in order to establish a tumor microenvironment that promotes tumor cell survival and dissemination. CAFs, cancer-associated fibroblasts.

EVs play an active role in the pathogenesis of neoplasia. Their role starts when normal cells exposed to a carcinogenic insult change the quantity and cargo of the released EVs. The cargo of these cells seems to primarily promote neoplastic transformation, the emergence of tumor-initiating cells, and cancer cell progression. Cancer cells release a variety of EVs, which are able to influence the behavior of recipient cells to promote cancer cell survival (Fig. 4). EVs participate in the horizontal transfer of biological information among not only cancer cells but also noncancerous cells both at the tumor niche and in distant organs where they contribute to the preparation of a permissive niche for metastasis. In addition to tumor-initiating cells and cancer cells, EVs can be produced and released by nonneoplastic cells residing in the organ where the primary tumor is located or even in distant tissues (the potential sites of future metastasis). These vesicles can either favor or impair tumor cell viability, proliferation, and invasion, actively contributing to the pathogenesis of cancer.^90^ EVs play a key role in modulating cancer immunity and in the crosstalk between tumor and immune cells. EVs represent a mechanism used by tumor cells to escape the host immune system.^10,71,193^ Knowledge of the role of EVs in cancer is mainly based on research done in vitro, or in induced mouse models, and the in vivo behavior of EVs in spontaneous cancer models remains to be elucidated due to the lack of reliable methods to visualize and detect EVs in diseased tissues. The following sections focus on the role of EVs in cancer initiation, growth, and progression.

### EVs in Pre-Neoplastic Lesions and Carcinogenesis

EVs have an active role in the multistep carcinogenesis process and actively contribute to cancer initiation and progression. Several known cancer risk factors have been linked to the release and uptake of EVs with specific cargo that can actively participate to cancer initiation. However, the number of published studies in this field is still limited. Cancer risk factors such as environmental chemicals (tobacco from cigarette smoking, arsenide),^65,209^ bacterial or viral infections,^123,124,180^ diet-related factors (obesity),^110^ hormonal factors (estrogen, androgen),^166,175^ and ultraviolet (UV) radiation^26^ have been linked to changes in EV release and contents, and could suggest that EVs play a role in cancer initiation. EVs seem to contribute to tumor development in 2 different ways: (1) by inhibiting the release from normal cells of EVs to carry tumor-suppressive mediators that disrupt cancer signaling pathways;^90,98^ and (2) by carrying an oncogenic content favoring tumor development and growth during exposure to risk factors such as chronic inflammation and environmental carcinogens.^98,108^ It has thus been demonstrated that EVs can both suppress and promote cancer initiation, through the action of different EV-associated proteins and miRNAs with opposing functions.

### EVs in Tumor Growth

One of the first recognized effects of EVs in tumors is that they induce or increase cell proliferation in recipient cells by the transfer of RNAs or proteins that are oncogenic or inhibit tumor suppressors. miRNAs have a role in modulating key pathways that induce cell proliferation.^187^ As an example, miR-222, which is overexpressed in melanoma cells and transferred to other melanoma cells via EVs, induced the activation of the PI3/AKT pathway in recipient cells.^57^ Osteosarcoma-derived EVs can contain miRNAs with oncogenic functions, such as miR-135, which is able to promote osteosarcoma cell proliferation, as well as invasion.^156^ Proteins can also be carried by EVs and are able to activate the same pathways inducing cell proliferation.^152^ As an example, in in vitro models of prostate cancer, tumor cell-derived EVs can contain the full-length androgen receptor protein, which can be transported to the nucleus of androgen receptor-null cells and enhance the proliferation of recipient cells in the absence of androgen.^166^ Tumor-derived EVs can also contribute to the inhibition of the apoptotic cell death by upregulating the expression of anti-apoptotic proteins such as Bcl-xL,^4^ survivin, XIAP, and cIAP1,2.^93,195^ Canine mammary tumor cell–derived EVs had higher levels of miR18a, miR19a, and miR181a compared to canine normal epithelial cell–derived EVs, and it is hypothesized that the miRNAs in cancer-derived EVs might regulate pathways that are important for mammary tumor maintenance and progression, such as cell division, antiapoptotic pathways, and hormone activity mediated by the estrogen receptor.^59^

### EVs in Cancer Progression

Numerous studies have demonstrated the relevant role of EVs in inducing a more malignant and aggressive phenotype in tumor cells. This is largely due to the modifications that tumor cell–derived EVs have on other tumor cells and the surrounding nonneoplastic cells (such as mesenchymal, endothelial, and immune cells) that form the neoplastic niche (Fig. 4). In addition, the surrounding nonneoplastic cells also produce EVs and are, in turn, able to influence and modify the behavior of neoplastic and nonneoplastic cells. Most of these changes are pro-tumoral; however, antineoplastic effects have also been described.^28^ Furthermore, *cancer stem cells* produce EVs and are strictly dependent on the intercellular communication with their stem cell niche.^13^ This is evident from several studies indicating that EVs derived from cancer stem cells are enriched with specific miRNAs and can induce activation and proliferation of several cell types, such as cancer-associated fibroblasts and tumor-associated macrophages.^174,176^ Finally, cancer cells that underwent *senescence or apoptotic cell death* (eg, in response to cancer drug treatments) can also release EVs thereby influencing the behavior of other neoplastic cells and neighboring nonneoplastic cells, favoring cancer progression.^189^

EVs secreted by tumor cells can be enriched with pro-angiogenetic factors, stimulating endothelial cell motility and vessel sprouting during *angiogenesis* to supply nutrients and oxygen to tumor cells.^34,51,69,70,216^ Finally, cancer cells produce more EVs under hypoxic conditions and hypoxia-induced EVs promote tumor angiogenesis, as well as invasion and metastasis.^178^

EVs affect *epithelial-to-mesenchymal transition*. This is a process by which epithelial tumor cells lose their differentiation and acquire a mesenchymal-like phenotype that renders them more invasive and promotes metastatic spread.^20,48^ Tumor cells undergoing epithelial-to-mesenchymal transition can produce EVs that induce epithelial-to-mesenchymal transition in neighboring tumor cells, as has been observed in prostate cancer^52^ and melanoma.^207^ Furthermore, EVs associated with epithelial-to-mesenchymal transition can promote fibroblast activation^50,74^ and myofibroblast phenotype differentiation,^32,204^ thereby contributing to the formation of *cancer-associated fibroblasts*. The crosstalk between resident fibroblasts and neoplastic cells has emerged as one of the key components in the creation of a pro-invasive condition in the tumor niche. EVs released by cancer-associated fibroblasts can transport molecules that promote epithelial-to-mesenchymal transition and induce phenotypic modifications in tumor cells, for example, inducing a stroma-like phenotype in recipient breast cancer cells (miR-21, -378e, and -143) or prostate tumor cells (miR-409), or promote increased anchorage-independent growth and expression of stem cell (Oct3/4, Nanog, Sox2) and epithelial-to-mesenchymal transition (Snail and Zeb) markers.^49,89^ In early cutaneous melanoma lesions (melanoma in situ), melanoma cells produce specific melanoma-associated vesicles, called melanosomes. These are normally involved in the production and transfer of melanin pigment to neighboring keratinocytes, but they are enriched with specific microRNAs, such as miR-211, able to induce features of cancer-associated fibroblasts in distant dermal fibroblasts before invasion of the melanoma.^50^ Cancer-derived EVs are also enriched in molecules (such as tetraspanins, adhesion molecules, and proteases) that mediate the digestion of or the interaction with extracellular matrix components,^137^ a step in cancer progression indispensable to the passage of tumor cells through the extracellular matrix.^21^ In this context, EVs from various cell types and body fluids contain matrix metalloproteinases.^181^

In recent years, a role for adipose cells and adipose tissue–derived mesenchymal stem cells in the modulation of tumor cell behavior has been recognized and shown to involve EV release and uptake. Adipocyte-derived EVs can induce contrasting effects on tumor cells depending on the type of tumor, either promoting proliferation, migration, and metastasis (as in melanoma and breast cancer), or inhibiting proliferative and promoting apoptosis (as in ovarian cancer). Notably, a potential effect of obesity in stimulating premetastatic niche formation in the liver has also been suggested. Furthermore, cancer cell-derived EVs also have an effect on the surrounding adipocytes, by inducing the conversion toward a cancer-associated adipocyte phenotype that in turn affects tumor cell behavior. EVs are thought to have a role in the crosstalk between tumor and adipose tissue,^184^ also in the context of obesity, which is known to be a risk factor for cancer progression.^110^

### EVs in Metastasis

Once tumor cells reach the vessel lumen to become circulating tumor cells and start their dissemination to distant organs, they become vulnerable to immune surveillance and require mechanisms of escape from immune-mediated elimination.^130^ EVs produced by both tumor cells and platelets^197,220^ within the vessel lumen seem to contribute to the protection of tumor cells, helping them to survive.

The ways in which EVs can contribute to the metastatic spread of tumor cells are numerous and occur during different phases of this multistep process.^1^ Therefore, research is necessary to clarify these mechanisms and understand how they can be therapeutically exploited. EVs participate in the creation of a “favorable metastatic niche”, which is called a “premetastatic niche” when induced by cancer-derived EVs that reach distant sites through the circulation, or an “active metastatic niche” when induced by EVs released by nonneoplastic cells in distant organs.^82,194^ In the multistep process of metastatic spread, cancer-derived EVs first permeabilize vessels, promoting vascular leakiness and allowing for EV diffusion, before being taken up by parenchymal cells at the future metastatic site.^82,157^ Even though the majority of cancer-derived EVs distribute to the bone marrow, there is evidence that specific cancer cell–derived EVs disseminate to organs that mirror the donor cancer-specific metastatic sites.^208^ These observations allow us to speculate that cancer-derived EVs arrive in the future metastatic site where they influence the resident cells to attract cancer cells. Indeed, cancer-derived EVs might be involved in controlling the organotropism of metastases. EVs show distinct integrin expression patterns that are related to the specific site of metastasis, suggesting that EV integrins could be used to predict organ-specific metastasis.^82^

Regional lymph nodes represent the first site of metastasis for many tumors, such as melanomas or carcinomas. In an early stage of melanoma, tumor-derived EVs efficiently disseminate via lymphatics, reaching regional tumor-draining lymph node, where they are more abundant compared to other organs and tissues.^163^ In the lymph node, during tumor progression or anticancer treatment, tumor-EVs induce a protumoral environment, interacting and altering the functions of nodal tumor suppressing cells (subcapsular sinus macrophages).^119,163^

The highly metastatic pancreatic ductal adenocarcinoma cells release EVs into the blood. Once these EVs reach the liver, they are taken up by resident histiocytic cells (Kupffer cells), inducing the formation of the liver premetastatic niche. Activation of Kupffer cells by EVs leads to a cascade of events with involvement of inflammatory cells that precedes the establishment of metastases.^36^ Furthermore, hepatic niche-derived (nontumor-derived) EVs can also have a role; for example, they can affect breast cancer cells by inducing changes consistent with a mesenchymal-to-epithelial transition,^47^ modulating the expression of key molecules such as E-cadherin and β-catenin.^47,105^ This is a process by which (epithelial) tumor cells that underwent epithelial-to-mesenchymal transition at least partially re-acquire their differentiated phenotype that creates in distant sites a tumor similar to the primary cancer.^112^

Bone is also a frequent site for metastasis of specific carcinoma types, such as breast and prostate cancers. Cancer cells can communicate with bone cells via EVs to enable the bone to permit cancer cell proliferation.^109,162^ Bone-tropic breast cancer cells secrete miR-218-enriched EVs. This specific miRNA directly and indirectly downregulated type I collagen expression by osteoblasts, inhibiting osteoblast differentiation and contributing to the adaption of the bone niche.^109^ Prostatic cancer cell–derived EVs enhanced osteoblast viability and produced a significantly more supportive growth environment for prostatic cancer cells when grown in co-culture with EV-treated osteoblasts.^162^

Once tumor cells arrive at the metastatic site they can change their phenotype through mesenchymal to epithelial transition, proliferate,^104^ or undergo dormancy and later reactivation. The potential role of EVs in the regulation of cancer cell dormancy at the metastatic sites has recently been recognized and has potential therapeutic applications. During the process of metastasis, not all cancer cells that reach the distant metastatic sites actually give rise to metastatic tumor growth. Some of them find an unfavorable environment in which factors secreted by local cells regulate the entry and retention of tumor cells into “cancer cell dormancy” and the forming of the so-called “sleepy niche.”^158^ EVs released by nonneoplastic cells in these sites participate in this process.^17,25,151^

## Fields of Application

**Figure 5.**
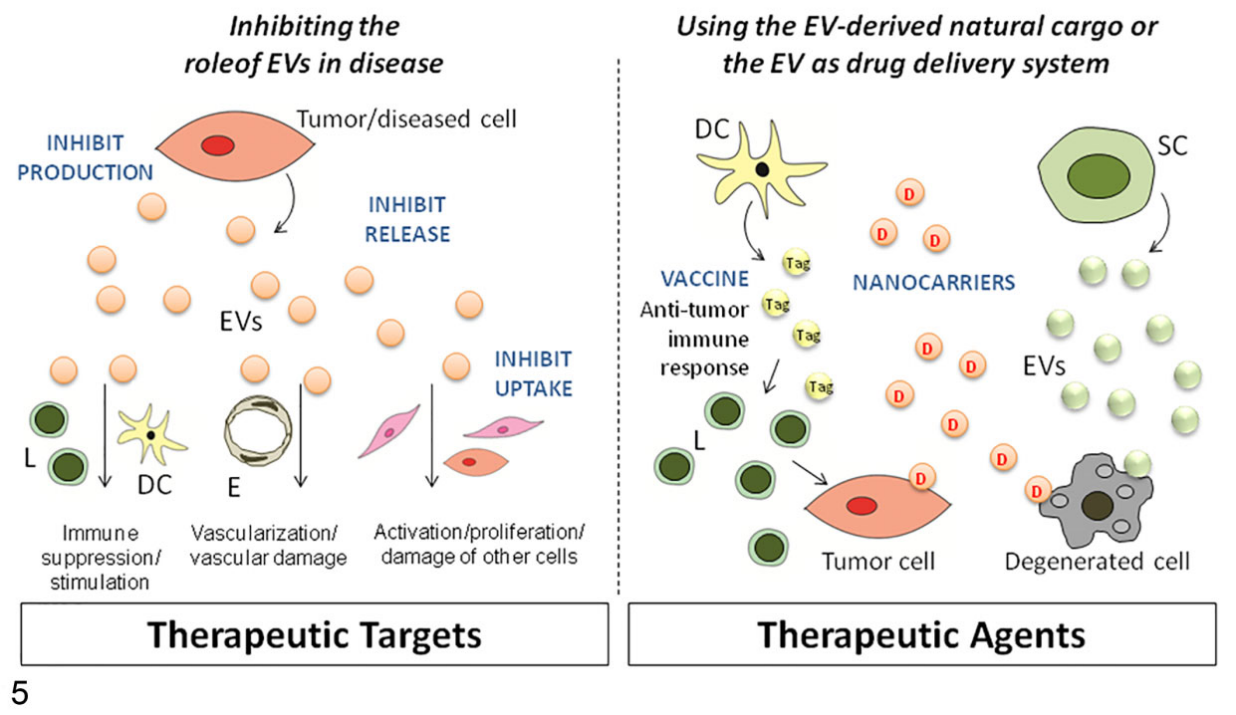
Extracellular vesicle (EV)-based therapy. EVs can be exploited both as therapeutic targets, to inhibit their biogenesis, release, and uptake, and as therapeutic agents, using them as vaccine or nanocarriers to transport therapeutic molecules. E, endothelial cells; L, lymphocytes; DC, dendritic cells; SC, stem cells; CAF, cancer-associated fibroblasts; Tag, tumor antigen; D, drug.

Understanding the mechanisms of action of EVs provides new insights into pathogenesis and may lead to the development of new therapies for cancer,^182^ degenerative diseases,^78^ and skin diseases.^120^ These innovative therapies are based on exploiting the cargo function of EVs to deliver drugs to target cells or blocking EV biogenesis, release, or uptake (Fig. 5). There are still limitations on developing EVs as a platform for drug delivery, mainly linked to the technology and lack of standardized methods for EV production and quality control. A few studies have been published on canine and swine models, mainly based on the use of EVs in regenerative medicine,^9,161,199^ and other reviews have been recently published.^88,173,182^ It is important to use the right disease models to test these new-generation drugs and this is an area where veterinary research can contribute.

Furthermore, many studies have aimed to unravel the molecular profile of EVs and to map the EV content alterations that occur within cells under the influence of disease, with the aim of discovering new reliable biomarkers.^218^ EVs could represent a rich and accessible source of biomarkers for cancer,^218^ inflammatory disorders,^35^ and diseases of the cardiovascular system,^46^ skin,^120^ kidney,^53^ liver,^12^ and nervous system^91,201,214^ in humans.

### Overview of EVs as Biomarkers

As EVs are released by diseased cells into the extracellular space and to the circulation, they can be isolated from many body fluids, and their molecular cargo can yield information about the cells of origin.^102^ This is the driving factor behind research into the use of EVs as biomarkers, predominantly in humans but increasingly also in veterinary medicine. Liquid biopsies are minimally or noninvasive compared to tissue biopsies. Liquid biopsies based on EVs are still in an early stage of development^177^ and there are only a few EV-based tests currently approved by the Food and Drug Administration, mainly for cancer patients.

EVs contain different cargo derived from their donor cells including proteins, lipids, and the nucleic acids: DNA, messenger RNA, small noncoding RNA, such as miRNA, and long noncoding RNA.^86,117,218^ These are candidate biomarkers because they reflect the state of the donor (diseased) cells at the time of formation, and this EV cargo can mirror variations in molecular expression over time. Several characteristics of EVs make it potentially advantageous to measure biomarkers in EVs rather than as free molecules in body fluids:

1. Biomarkers contained within EVs are more stable as they are shielded by the lipid bilayer from enzymatic degradation by (ribo)nucleases, proteases, and lipases, and from environmental and storage conditions, such as freezing, thawing, and pH.^218^
2. Biomarkers may be enriched in these vesicles; that is, present in a higher concentration and therefore more readily detectable than in body fluids.

Thus, capturing of these small satellites of information from the blood (or other fluids) may give us a glimpse of what is going on in diseased tissues.

### EVs as Cancer Biomarkers

In the field of cancer research, tumor-derived EVs and their cargo may have utility in early cancer detection, diagnosis of cancer, assessment of prognosis, predicting response to cancer therapy, and monitoring during treatment. Tumor-derived EVs are a potentially rich and accessible source of cancer biomarkers that can be obtained from the minimally or noninvasive liquid biopsy,^102^ whereas solid tissue samples are a more invasive and sometimes dangerous—particularly in the case of tumors in the brain and central nervous system—source of EVs.^102,201,205^ EVs produced by tumor cells in the central nervous system can be detected in the circulation, since they are able to cross the brain-blood barrier,^135^ and in cerebral-spinal fluid where EVs from patients with glioblastoma contain more miR-21 than those from healthy humans.^179^

*EV-derived DNA* is the basis of the first EV-based tests. Two examples are ExoDx Prostate, aimed at detecting a combination of specific mutations in EVs isolated from urine samples for the diagnosis of high-grade prostate carcinoma;^121^ and ExoDx Lung, based on the detection of EGFR mutations in circulating EVs.^27^ Indeed, EVs can be a source of cancer-derived DNA, including mitochondrial DNA and large fragments of dsDNA, which can be used to detect mutations of the tumor cells that produced the EVs.^194^

*EV-derived RNA* including *miRNA* is a potential diagnostic biomarker. miRNAs form a minority of noncoding RNAs present in EVs, with vault RNA, Y-RNA, and specific tRNAs being among the most abundant small noncoding RNAs.^147^ Nevertheless, EV-derived miRNA may be good tumor biomarker candidates.^60,148^ Importantly, not all circulating miRNAs are within or associated with EVs, since miRNAs can circulate bound to proteins or may be released from circulating tumor cells and blood cells. Thus, for analysis of EV-derived miRNAs, it is fundamental to isolate EVs first and then analyze their miRNA cargo. Using this approach on plasma-derived EV samples, miRNAs participating in tumor invasion and metastasis such as miR-21, miR-10b, miR-19a, miR-105, miR-122, and miR-223 have been identified as prognostic biomarkers for metastatic lung and breast cancer.^107,221^ As an early cancer diagnostic tool, acute myeloid leukemia-derived EVs with a set of specific miRNAs were detected in circulation prior to the appearance of leukemic blast cells in the blood.^81^ Furthermore, a specific miR signature in circulating EVs established the diagnosis of pancreatic ductal adenocarcinoma and differentiated it from chronic pancreatitis, and was superior to EV protein GPC1 (glypican-1) or plasma CA (carbohydrate antigen) 19-9 levels, the only Food and Drug Administration–approved biomarker for management of pancreatic ductal adenocarcinoma.^101^

*Messenger RNA (mRNA), long RNA, and circRNA* are also present within EVs.^117,165^ In particular, EV-associated long RNA in the circulation reflected the tissue origins and the relative fractions of different immune cell types. Furthermore, their profiles could distinguish patients with cancer from healthy individuals.^106^

*Protein cargo* of EVs derived from liquid biopsies is another potential biomarkers, particularly for early cancer detection.^41^ Glypican-1, a surface protein in circulating EVs, was elevated before pancreatic cancer was detectable by imaging techniques and was highly expressed in patients at an early phase of the disease compared to healthy humans.^125^ Furthermore, in early phases of breast cancer, circulating EV-derived survivin^92^ and fibronectin^134^ showed high levels of expression. Carcinoembryonic antigen from circulating EVs has shown better predictive value with higher sensitivity for metastatic colorectal cancer compared to serum levels of the same protein.^213^ Furthermore, EV-derived molecules may be useful in selecting patients for therapy. For example, HER2 levels in EVs derived from the plasma of breast cancer patients correlated with the levels in tumor tissue biopsies.^55^

In *dogs*, a few articles show the potential utility of EVs as biomarkers. Information on the selection of reference genes for miRNAs isolated from circulating EVs is available.^142^ Different techniques to isolation and detection EVs in blood samples have been described.^3^ Other studies compared the concentration of blood EVs in healthy dogs to those in dogs with different types of tumors,^221^ mast cell tumor,^183^ or osteosarcoma,^22^ but these studies had contrasting results. The applicability of EVs as cancer biomarkers in dogs is an almost completely unexplored field of research with great potential.

### EVs as Biomarkers of Other Diseases

EV-derived cargo has been investigated as a biomarker for other types of diseases, such as human,^46,143,168^ canine,^212^ and feline^38^ cardiovascular diseases, and metabolic disorders of humans^26,29,145^ and cows. Indeed, most work in veterinary medicine on EVs as biomarkers has been in *dairy cattle*. In particular, different methods have been applied and compared for detection of circulating EVs^97,217^ to monitor the metabolic state, pregnancy,^115,160^ or uterine infections.^6,7^ Changes occur in EVs as postpartum dairy cows adapt to lactation,^5,39,40^ suggesting that circulating EVs might be used as biomarkers of metabolic disease risk.^5^ The potential utility of EVs in the diagnosis of bovine tuberculosis was demonstrated using an in vitro model in which the EV-derived lipoprotein LpqH was used to distinguish between paratuberculosis infection or vaccination against tuberculosis infection.^153^ In *horses*, circulating EVs were explored as diagnostic markers for equine regenerative anemia^169^ or laminitis in ponies,^58^ based on their specific protein cargo or antigens that reflected their cell of origin, respectively. EVs were isolated from *synovial fluid*, but no differences were found in their concentration among horses of different ages.^19^

Easily accessible sources of EVs have been used for diagnostic purposes, including *urine-derived EVs* for human urological cancer,^44,202^ and potentially for other nonurological malignancies.^54^ In dogs and cats, urinary EV-derived specific miRNAs isolated from fresh urine samples reflected changes in renal function, as they were differentially expressed in both cats and dogs with high levels of serum urea nitrogen and creatinine, as well as in dogs with higher histological “kidney damage score,” compared with healthy control animals with normal renal function. These data suggested urine EVs and their cargo as potential biomarkers for the diagnosis of kidney disorders in animals.^84,85^

Furthermore, *salivary EVs* are potential biomarkers of human oro-pharyngeal cancers,^141^ and perhaps also for systemic diseases^76^ such as diabetes during early pregnancy.^133^ Human *breast milk* also represents a biofluid from which EVs can be detected and analysed.^75,222^ Studies in dairy cows compared different methods for EV isolation from milk.^172,186,196,211^ Milk EVs from cows that were uninfected or infected with *Staphylococcus aureus* had differences in specific miRNAs (bta-miR-142-5p and -223) that are potential biomarkers for the early detection of bacterial infection of the mammary gland.^186^

### Current Limits in EVs Application as Biomarkers

It is straightforward to isolate EVs from blood and other body fluids, but there are still limitations to the routine application of EV-based testing in the clinic,^218^ including a lack of standardization of methods for isolation, detection, and analysis of EVs.^191^ Currently, EVs are isolated based on their physiochemical characteristics, as briefly explained in the following section. However, for use as disease biomarkers, it may be more sensitive to isolate and identify cell type–specific and cell status–specific EVs from circulation by measuring panels of cell-surface differentiation antigens shared by EVs and their donor cells. However, the small size and high heterogeneity of EVs make this a difficult undertaking. New isolation methods, such as the use of DNA-assisted immunoassays, have been investigated to detect the scarce EV subpopulations that have the same surface markers as diseased or neoplastic cells.^103,206^

## Methods for Isolating and Detecting EVs

### Isolating EVs

**Figure 6.**
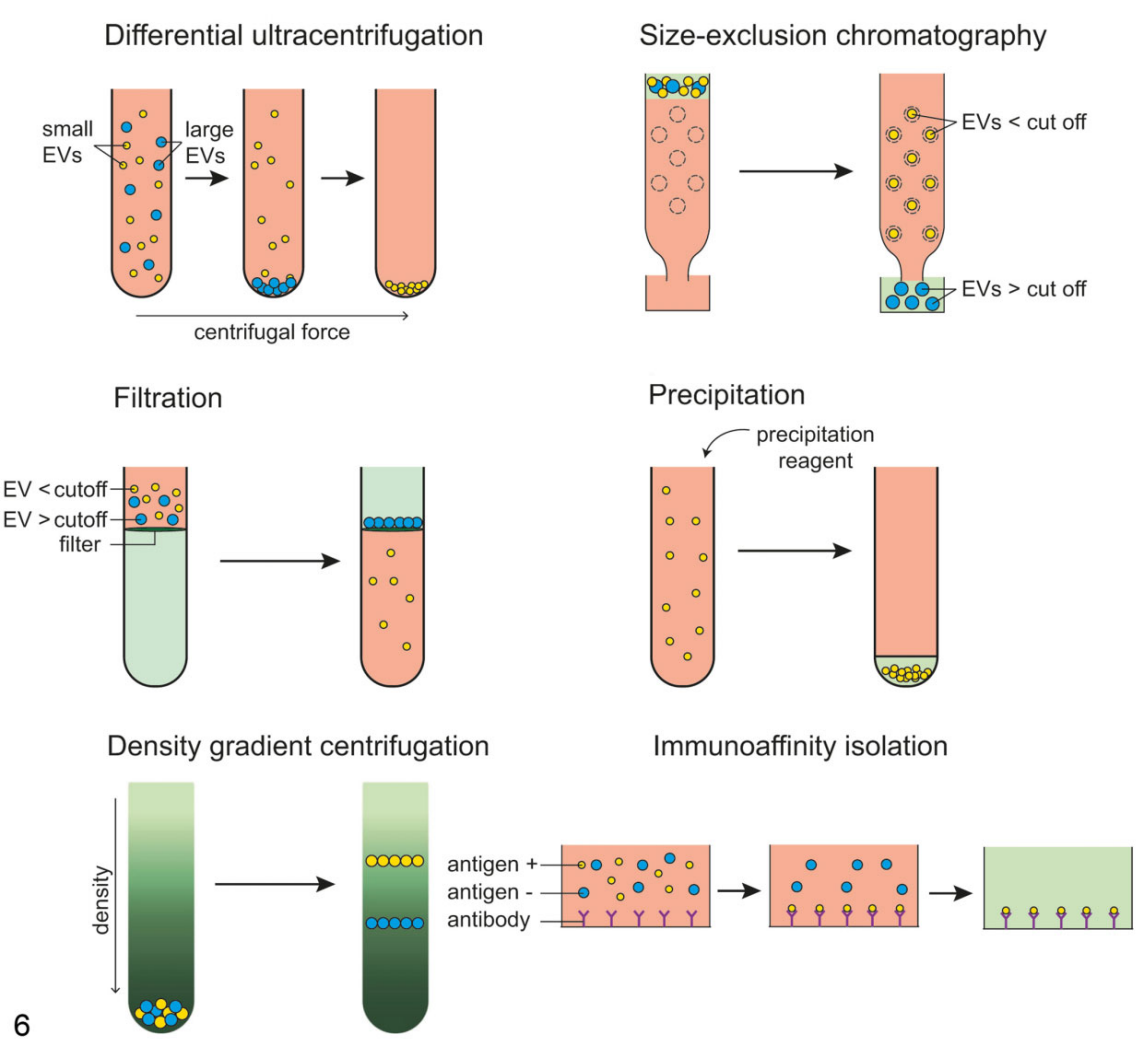
Most frequently used methods for the isolation of extracellular vesicles (EVs). The isolation of EVs can be based on size, buoyant density, or by detecting an antigen. Differential ultracentrifugation uses centrifugal force to separate EVs based on size, as larger EVs collect earlier and at a lower centrifugation speed compared to small EVs. In size-exclusion chromatography, a column with a porous matrix is used to separate EVs by size. Filtration concentrates EVs in a sample by passing them through a filter. In precipitation, a reagent is added to a sample concentrate EVs in a pellet. In density gradient centrifugation, EVs are separated into specific layers of a density gradient, as they settle in the layer with their equilibrium density. The immunoaffinity isolation method uses antibodies to capture EVs based on their antigenicity.

Several methods have been developed for the isolation of EVs from biological fluids or conditioned cell culture medium, although the efficacy and the purity of EV preparations varies. Each of these methods uses the biophysical and biochemical characteristics of EVs, such as mass density, size, and shape, to separate them from other particles. One routinely applied technique is differential centrifugation, which applies stepwise increases in centrifugal force to pellet particles based on their density and size (Fig. 6). This separation of EVs into apoptotic bodies (pellet at 2000 × *g*), large EVs (pellet at 5000-10 000 × *g*), and small EVs (pellet at ≥100 000 × *g*). The main limitation of differential centrifugation is that the resulting EV pellets contain a significant amount of contaminants such as proteins. Furthermore, this method of EV isolation is time-consuming, hampering its application in the clinic.

Other, less time-consuming EV isolation methods are also employed in EV research. These include size exclusion chromatography, ultrafiltration, and precipitation (Fig. 6). These have reduced assay time compared with differential centrifugation, but still the EV preparations may contain significant amounts of contaminants affecting the quality of the samples. Size exclusion chromatography uses a column containing an exclusion matrix to separate particles based on size.^37^ Consequently, particles that are larger than the pore size of the exclusion matrix, such as protein aggregates, will co-isolate with EVs. In ultrafiltration, EVs are separated from soluble components by passing the sample through a filter.^37^ EVs and other particles larger than the pores in the filter are retained on the filter, while smaller components pass through. Larger particles, such as protein aggregates, co-isolate with EVs and are the major contaminants in EV samples prepared by ultrafiltration. Precipitation makes use of differential solubility of components in polyethylene glycol. This method has the highest level of contaminants, such as proteins including albumin, apolipoprotein E, and other lipoproteins, and Tamm Horsfall protein (from urine). Moreover, residual polymer structures in the isolated EV preparations may hamper structural and functional analysis. Based on the authors’ experience, differential centrifugation isolates slightly more pure EV populations, even if the separation is mainly based on size (and partly on density). Therefore, EV preparations sedimented by differential centrifugation are also contaminated with larger protein complexes. When used for fluids rich in lipoprotein particles (eg, plasma), an advantage over precipitation is that with differential centrifugation, many (but not all) of the lighter and smaller lipoprotein particles (eg, very low density lipoprotein, chylomicrons, high-density lipoproteins) are discharged.

A more robust procedure for the isolation of EVs is density gradient centrifugation (Fig. 6), which is often performed to further purify EV preparations after other initial enrichment procedures (eg, differential centrifugation or size exclusion chromatography). In density gradient centrifugation, EVs are loaded in sucrose or iodixanol density gradients and centrifuged until they reach their equilibrium densities. Density gradient centrifugation establishes a separation based on size and mass density or mass density only, depending on whether a top-down gradient or bottom-up gradient is used.^37^ The major advantage of density gradient centrifugation is that it separates EV from proteins and therefore results in a purer EV preparation compared with the other methods. However, contaminants such as high-density lipoproteins can still be included in the sample if they have similar buoyant densities as EVs. Density gradient centrifugation gives low EV recovery compared to the other methods and is time-consuming, which prohibits its use in a clinical setting.

Immunoaffinity isolation techniques can be used to isolate specific subpopulations of EVs (Fig. 6). These assays use surfaces coated with antibodies that bind to ligands, often proteins, on the EV surface. Specific subpopulations of EVs that have surface expression of the target antigen can be isolated. Immunoaffinity-based assays can be readily made in the form of a chip or plate and are therefore applicable in a clinical setting.

The EV isolation method of choice depends on the type of body fluid that is analyzed, the research question, and the downstream analyses required to answer the research question. For EV-associated biomarkers of disease, contaminants interfere with accurate interpretation of the results. To prove that disease biomarkers are associated with EVs, it is crucial that EV preparations are as pure as possible. However, methods giving highly pure EV preparations often have lower EV yields; effectively, EV-based biomarker research constantly faces a trade-off between EV purity, clinical applicability, and biomarker yield. A great challenge in this field is the variability of protocols used in different clinical studies to isolate or enrich for EVs, which probably give rise to a much variability in the data.

### Analyzing EVs and Their Biomolecular Cargoes

To date, there is no gold standard technique for the quantification of EVs, since no single method can analyze the full spectrum of EV properties in biological fluids or clinical samples. The majority of EV-related studies used several complementary approaches to analyze EVs.^79,188^ In general, these methods are based on physical or biochemical analyses to identify and quantify EVs in a sample. Additionally, EV biomolecular cargoes are investigated, for example, using proteomics, genomics, and lipidomics methods.^113^ The main features of the most used methods for the analysis of EVs are described below and detailed information is provided by MISEV2018.^191^

#### Analysis Based on EV Physical Properties

Analyses of physical properties include electron microscopy and nanoparticle tracking analysis. These methods determine EV diameter directly via high-resolution imaging, or indirectly using indirect electrical readouts. High-resolution flow cytometry is a further method allowing the analysis of EVs based on physical properties that requires advanced equipment and is therefore not widely applied.

Electron microscopy allows visualization of the morphology and size of EVs. Electron microscopy has recently been applied to characterize EVs of canine and feline mammary cancer cells. The same study also used transmission immuno-electron microscopy using immunogold-labeled antibodies to common EVs markers (CD63 and Alix).^171^ A limitation of this technique is the limited number of EVs that can be analyzed. For this reason, it is impossible to use electron microscopy to analyze a representative population of EVs that vary in size and composition, as is present in biological and clinical samples. Related techniques that can be used to analyze EVs are scanning electron microscopy, cryo-electron microscopy, and scanning-probe microscopy including atomic force microscopy.^191^

Flow cytometry is commonly used for the analysis of cells and has been adapted for the analysis of EVs. High-resolution flow cytometry, which can detect particles smaller than ∼500 nm, is one of the best techniques for EV quantification and enumeration. It uses light scattering and fluorescence parameters, which may include labeling of specific EV components.^145,200^ However, there is still the risk of swarm effect when the sample is too concentrated (multiple EVs are simultaneously illuminated by the laser and counted as a single particle), so it is crucial that samples are diluted until single EV measurements are reached.^185^

Nanoparticle-tracking analysis estimates the diffusion coefficient and size of individually observed EVs by analyzing their motion trajectories. One major limitation of this technique is that size determination could be less accurate, particularly for populations of smaller particles.^154^ This might be even more complicated for analyzing EV concentrations in complex biofluids, because protein aggregates and lipoproteins may be other sources of scattering.^122^ Nonetheless, despite these limitations, nanoparticle-tracking analysis represents one of the most used methods for fast assessment of both size distribution and concentration of EVs.

Dynamic light scattering is another technique used to analyze EVs. It analyzes the scattering of a laser beam, but with limitations on the smallest size of detectable particles (ranging from 1 nm to 6 μm) and reduced accuracy in suspensions of particles varying in size.^191^

#### Analysis Based on EV Biochemical Properties

*Biochemical analyses* can be performed on EV samples to confirm the presence of EVs or to select a specific subclass of EVs. These assays can only be used for measuring highly purified EV samples, since measurements can be compromised by protein contamination of the sample. The most commonly used biochemical methods can be divided into conventional protein analysis (immunoblotting assays) and assays that capture specific EVs (immunosorbent EV assays).

Immunoblotting of specific proteins is used to confirm the presence of EVs in a sample, or determine their cell of origin or protein composition. Purified EVs are lysed to solubilize EV proteins, which are directly spotted on a membrane (in a dot blot assay) or separated using SDS-PAGE (in a Western blot assay) and the protein of interest is detected with labelled antibodies. Based on the MISEV2018 guidelines, the presence of EVs should be demonstrated by detecting at least one transmembrane protein associated with the plasma membrane (such as CD9, CD63, CD81) and one cytosolic protein in EVs (eg, TSG101, ALIX).^191^ Immunoblotting is most frequently used for this purpose. Immunoblotting can also be used for the detection of EV-associated proteins, such as for use as biomarkers of disease. However, it is important to note that immunoblotting is a semiquantitative method and that as for other bulk assays it does not provide any information about EV heterogeneity.

Immunosorbent EV assay is a modification of the classical enzyme-linked immunosorbent protein assay in which antibodies with affinity for EV membrane proteins are used to capture and detect specific EVs. A variant of this method uses magnetic beads for EV capture. This has a potentially increased capture efficiency because of the mobile capture surface. These assays have proved to be valuable when used with other techniques such as flow cytometry for quantification of EV surface proteins in complex samples such as urine or blood without prior EV isolation and purification. However, it must be realized that so far, no protein targets have been identified that cover the full spectrum of EVs or are present on every vesicle within an EV population. Thus, these approaches only quantify the EV-associated target proteins and the selection and use of specific antibodies implies that only a specific subset of EVs carrying the targeted proteins will be characterized. The future clinical application of this method relies on ongoing research to identify disease-specific panels of antigens on the EV surface that are shared by diseased donor cells and their released EVs. This would allow the specific detection and quantification of EVs produced and released by the diseased cells.^79^

The major challenges in the analysis of EVs is their heterogeneity in size and composition, and the difficulty in distinguishing EVs from similarly sized structures such as lipoproteins, protein aggregates, and viruses.^113^ To date, no single technology can perform the full range of EV analyses making a combined approach the only option for comprehensive EV analysis at this time. Furthermore, there are still several limitations before use of EVs will be possible; these are linked to the need for standardization of analytical procedures in order to generate comparable results. This renders the field of EV research an exciting and challenging new domain for clinicians and scientists alike.

## Contribution of Pathologists to Studying EVs Secreted by Diseased Tissues

### Limitations of Current Methods for Studying EVs in Diseased Tissues

To date, only a few articles have been published on how to isolate EVs directly from solid tissues.^83,198^ In these studies, EV were isolated from brain tissue by creating a tissue cell suspension that was subjected to centrifugation and ultracentrifugation steps followed by sucrose or iodixanol density-gradient flotation. Gentle manipulation of the tissue to avoid cell damage is essential to obtain reliable results, as is prompt freezing at −80 °C for storage. Postmortem delay, longer storage time, and freeze-thaw cycles will negatively impact the tissue quality and result in contamination of the fractions with cellular debris and vesicles that were not actively released by cells. This method seems to be promising and applicable to other tissue types. Indeed, EVs were isolated from lymph node and skin melanoma metastases using a similar centrifugation-based protocol,^87^ which was combined with electron microscopic analysis of tumor tissue to visualize EVs in the interstitial space of melanoma metastases. Visualization of EVs within intact diseased tissue would be extremely interesting, but reliable methodology is currently lacking to perform such experiments.

Several authors have tried to observe and quantify the immunohistochemical expression of EV markers in human tumor tissue samples. However, some known EV markers, such as CD63, are also expressed by cells. Thus, although increased CD63 expression in gastric cancer cells is suggested as a prognostic factor,^126^ it cannot be directly linked to the functions of EVs released by gastric cancer cells. There are several identified EV markers (summarized in a detailed table in MISEV2018)^191^ that are generic or specific for the tissue of origin, and they are usually used when EVs have already been isolated from body fluids or culture media, to confirm the presence of EVs or to isolate and analyze a specific subgroup of EVs. However, the fact that these markers are shared by EVs and their donor cells make them inappropriate to identify EVs directly in tissue, for example, by the use of immunohistochemistry.

It would be a major advance to be able to isolate and quantify EVs and analyze their cargo in the context of their tissue location. This would identify exactly where they are concentrated in the tissue, and clarify their physical relationship to the donor and recipient cells within the diseased tissue. For example, a tumor mass is considered to contain neoplastic cells that are particularly active in communicating with their environment: neoplastic cells that are at the invasive front, or close to vessels, or aggregated in a small group in the so-called “collective cancer invasion.” Thus, these cells are most likely to actively produce EVs in order to communicate with neighboring mesenchymal, endothelial, and immune cells, to thereby facilitate their own migration. One possible approach would be to isolate EVs from solid tissue^83,198^ with the use of EV markers and tissue microdissection techniques, in order to colocalize the EV cargo produced by the tumor cells with their specific distribution in the tissue, to create a *topographic EV characterization* of tumor tissue.

### Roles of Pathologists in Studying the Role of EVs in Pathogenesis and Disease Progression

During in vitro functional studies on the role of EVs in diseases and cell-to-cell interactions, molecules such as miRNA and proteins are identified within EVs isolated from the conditioned medium of donor cell lines. Several of these molecules have been shown to have an effect on recipient cells when these cells are cultured with EVs. However, little is known about the functional importance of EVs in vivo or more importantly in diseased tissues. Histopathological analysis of the diseased tissues is therefore essential to understand the pattern of expression of the identified molecules, known to be produced by some specific cell types. For example, specific neoplastic cell subpopulations exist within a tumor and these might produce EVs with different cargoes. These, in turn, may be able to differentially change the phenotype and influence the behavior of other tumor cells or cells in the tumor microenvironment. The contribution of all these different EV cargoes are likely to be to tumor progression, invasion, and metastatic spread. Pathologists may be able to help EV scientists to understand which EV cargoes are produced by which subpopulation of tumor cells: apoptotic tumor cells,^155^ senescent tumor cells,^189^ cancer stem cells,^34,70^ and drug-resistant tumor cells^18^ are just some examples of these neoplastic subpopulations.

Identification of the recipient cells is also crucial to understand the involvement of EVs in pathogenesis and disease progression, but still represents a very challenging issue in the field. First, there must be clear evidence that a change in a cell’s behavior is a direct result of the binding or uptake of EVs. However, it is not easy to accurately identify cells that have been targeted by an EV based on currently available methods. Lipid dyes, for example, are used to stain EVs for targeting experiments, but these dyes are not EV-specific and can become separated from the EVs, be internalized by cells, or stay in tissues even after EVs are degraded or internalized by the cells, leading to inaccurate interpretation of results.^66^ It is also challenging to exactly pinpoint which of the many molecules associated to EVs are causing an effect on target cells. New methods are needed to prove that EV-associated molecules do induce changes in a recipient cell.

### Roles of Pathologists in Studying Biomarkers in Liquid Biopsies

There are numerous tissue markers but far fewer circulating markers that are useful for diagnosis, prognosis, or predicting therapeutic efficacy. Pathologists can use their knowledge of tissue biomarkers to suggest potential EV-related biomarkers for further investigation. Since the content of EVs tends to mirror that of their donor cells, EVs might express disease-specific markers as well as differentiation markers of the cell of origin; this knowledge could be applied to techniques for isolation and identification of EVs. Furthermore, since EVs can be purified, their numbers and contents can be enriched manifold. Consequently, markers that were previously too diluted to be measured in biological fluids could be investigated.^46^

The contribution of pathologists to EV research is essential for the identification of novel, specific tissue and disease markers to be detected in EVs. They can also play a role in comparing the presence of the relevant markers in EVs from body fluids with expression and distribution of these markers in the diseased tissue, with prognostic histological features of a disease, histological grading systems, and with clinical data and follow-up information.

## Conclusion

This article reviews recent advances in the field of EV research in both the human and veterinary medical fields. What emerges is the important role of EVs and their cargo in the progression of diseases, and the enormous potential for using their biomolecular cargo as disease biomarkers or therapeutic tools. While small steps are being made toward the application of EV science to veterinary clinical medicine, for example, as biomarkers in canine, bovine, and equine medicine, more work is required to uncover their potential. Preliminary data suggest that circulating EVs could be of help in the diagnosis of cancers and infectious diseases in these species. Also, the analysis of EVs from alternative sources in animals, such as urine, milk, synovial fluid, shows potential and should be further investigated.

Unraveling the role of EVs in the pathogenesis of diseases of domestic animals will open new therapeutic possibilities and form the basis for innovative EV-based approaches that could subsequently be translated to human medicine. Preliminary data on the use of EVs as a form of tumor “vaccine” as well as in regenerative medicine are very promising.

EV research is a relatively new field, and EVs show great clinical potential for use in diagnosis and therapy. With their expertise in tissue biomarkers and the analysis of diseased tissues, there is a clear role for the pathologist in EV research as it characterizes the functions and clinical applications of these membrane-bound messengers.

## References

[1] Adem B Vieira PF Melo SA. Decoding the biology of exosomes in metastasis. Trends Cancer. 2020;6(1):20–30. 31952777 10.1016/j.trecan.2019.11.007

[2] Admyre C Bohle B Johansson SM, et al. B cell-derived exosomes can present allergen peptides and activate allergen-specific t cells to proliferate and produce TH2-like cytokines. J Allergy Clin Immunol. 2007;120(6):1418–1424. 17868797 10.1016/j.jaci.2007.06.040

[3] Aguilera-Rojas M Badewien-Rentzsch B Plendl J, et al. Exploration of serum- and cell culture-derived exosomes from dogs. BMC Vet Res. 2018;14(1):179. 29884196 10.1186/s12917-018-1509-x PMC5994050

[4] Al-Nedawi K Meehan B Micallef J, et al. Intercellular transfer of the oncogenic receptor EGFRvIII by microvesicles derived from tumour cells. Nat Cell Biol. 2008;10(5):619–624. 18425114 10.1038/ncb1725

[5] Almughlliq FB Koh YQ Peiris HN, et al. Effect of exosomes from plasma of dairy cows with or without an infected uterus on prostaglandin production by endometrial cell lines. J Dairy Sci. 2017;100(11):9143–9152. 28865856 10.3168/jds.2017-13261

[6] Almughlliq FB Koh YQ Peiris HN, et al. Proteomic content of circulating exosomes in dairy cows with or without uterine infection. Theriogenology. 2018;114:173–179. 29631032 10.1016/j.theriogenology.2018.03.024

[7] Almughlliq FB Koh YQ Peiris HN, et al. Circulating exosomes may identify biomarkers for cows at risk for metabolic dysfunction. Sci Rep. 2019;9(1):13879. 31554846 10.1038/s41598-019-50244-7 PMC6761115

[8] Antonucci F Turola E Riganti L, et al. Microvesicles released from microglia stimulate synaptic activity via enhanced sphingolipid metabolism. EMBO J. 2012;31(5):1231–1240. 22246184 10.1038/emboj.2011.489 PMC3297996

[9] Bach F Libregts S Creemers L, et al. Notochordal-cell derived extracellular vesicles exert regenerative effects on canine and human nucleus pulposus cells. Oncotarget. 2017;8(51):88845–88856. 29179481 10.18632/oncotarget.21483 PMC5687651

[10] Bae S Brumbaugh J Bonavida B. Exosomes derived from cancerous and non-cancerous cells regulate the anti-tumor response in the tumor microenvironment. 2018;9(3–4):87–100. 10.18632/genesandcancer.172 PMC6086005 30108680

[11] Bakhti M Winter C Simons M. Inhibition of myelin membrane sheath formation by oligodendrocyte-derived exosome-like vesicles. J Biol Chem. 2011;286(1):787–796. 20978131 10.1074/jbc.M110.190009 PMC3013037

[12] Balaphas A Meyer J Sadoul R, et al. Extracellular vesicles: future diagnostic and therapeutic tools for liver disease and regeneration. Liver Int. 2019;39(10):1801–1817. 31286675 10.1111/liv.14189

[13] Batlle E Clevers H. Cancer stem cells revisited. Nat Med. 2017;23(10):1124–1134. 28985214 10.1038/nm.4409

[14] Bianchi E Doe B Goulding D, et al. Juno is the egg Izumo receptor and is essential for mammalian fertilization. Nature. 2014;508(7497):483–487. 24739963 10.1038/nature13203 PMC3998876

[15] Bianco F Perrotta C Novellino L, et al. Acid sphingomyelinase activity triggers microparticle release from glial cells. EMBO J. 2009;28(8):1043–1054. 19300439 10.1038/emboj.2009.45 PMC2664656

[16] Biró É Sturk-Maquelin KN Vogel GMT, et al. Human cell-derived microparticles promote thrombus formation in vivo in a tissue factor-dependent manner. J Thromb Haemost. 2003;1(12):2561–2568. 14738565 10.1046/j.1538-7836.2003.00456.x

[17] Bliss SA Sinha G Sandiford OA, et al. Mesenchymal stem cell-derived exosomes stimulate cycling quiescence and early breast cancer dormancy in bone marrow. Cancer Res. 2016;76(19):5832–5844. 27569215 10.1158/0008-5472.CAN-16-1092

[18] Boelens MC Wu TJ Nabet BY, et al. Exosome transfer from stromal to breast cancer cells regulates therapy resistance pathways. Cell. 2014;159(3):499–513. 25417103 10.1016/j.cell.2014.09.051 PMC4283810

[19] Boere J van de Lest CHA de Grauw JC, et al. Extracellular vesicles in synovial fluid from juvenile horses: No age-related changes in the quantitative profile. Vet J. 2019;244:91–93. 30825901 10.1016/j.tvjl.2018.12.010 PMC7116028

[20] Bongiovanni L D’Andrea A Romanucci M, et al. Epithelial-to-mesenchymal transition: immunohistochemical investigation of related molecules in canine cutaneous epithelial tumours. Vet Dermatol. 2013;24(1):195–203.e42–e43. 23331698 10.1111/j.1365-3164.2012.01116.x

[21] Brachelente C Cappelli K Capomaccio S, et al. Transcriptome analysis of canine cutaneous melanoma and melanocytoma reveals a modulation of genes regulating extracellular matrix metabolism and cell cycle. Sci Rep. 2017;7(1):6386. 28743863 10.1038/s41598-017-06281-1 PMC5526991

[22] Brady JV Troyer RM Ramsey SA, et al. A preliminary proteomic investigation of circulating exosomes and discovery of biomarkers associated with the progression of osteosarcoma in a clinical model of spontaneous disease. Transl Oncol. 2018;11(5):1137–1146. 30053712 10.1016/j.tranon.2018.07.004 PMC6077151

[23] Brill A Dashevsky O Rivo J, et al. Platelet-derived microparticles induce angiogenesis and stimulate post-ischemic revascularization. Cardiovasc Res. 2005;67(1):30–38. 15878159 10.1016/j.cardiores.2005.04.007

[24] Carossino M Dini P Kalbfleisch TS, et al. Downregulation of Microrna eca-mir-128 in seminal exosomes and enhanced expression of CXCL16 in the stallion reproductive tract are associated with long-term persistence of equine arteritis virus. J Virol. 2018;92(9):e00015–18. 29444949 10.1128/JVI.00015-18 PMC5899189

[25] Casson J Davies OG Smith CA, et al. Mesenchymal stem cell-derived extracellular vesicles may promote breast cancer cell dormancy. J Tissue Eng. 2018;9:2041731418810093. 30627418 10.1177/2041731418810093 PMC6311537

[26] Castaño C Novials A Párrizas M. Exosomes and diabetes. Diabetes Metab Res Rev. 2019;35(3):e3107. 30513130 10.1002/dmrr.3107

[27] Castellanos-Rizaldos E Grimm DG Tadigotla V, et al. Exosome-based detection of EGFR T790M in plasma from non–small cell lung cancer patients. Clin Cancer Res. 2018;24(12):2944–2950. 29535126 10.1158/1078-0432.CCR-17-3369

[28] Chen X Chen XG Hu X, et al. MiR-34a and miR-203 inhibit survivin expression to control cell proliferation and survival in human osteosarcoma cells. J Cancer. 2016;7(9):1057–1065. 27326248 10.7150/jca.15061 PMC4911872

[29] Chen Y Li G Liu ML. Microvesicles as emerging biomarkers and therapeutic targets in cardiometabolic diseases. Genomics Proteomics Bioinformatics. 2018;16(1):50–62. 29462670 10.1016/j.gpb.2017.03.006 PMC6000161

[30] Chivet M Javalet C Laulagnier K, et al. Exosomes secreted by cortical neurons upon glutamatergic synapse activation specifically interact with neurons. J Extracell Vesicles. 2014;3:24722. 25398455 10.3402/jev.v3.24722 PMC4232649

[31] Choudhuri K Llodrá J Roth EW, et al. Polarized release of T-cell-receptor-enriched microvesicles at the immunological synapse. Nature. 2014;507(7490):118–123. 24487619 10.1038/nature12951 PMC3949170

[32] Chowdhury R Webber JP Gurney M, et al. Cancer exosomes trigger mesenchymal stem cell differentiation into pro-angiogenic and pro-invasive myofibroblasts. Oncotarget. 2015;6(2):715–731. 25596732 10.18632/oncotarget.2711 PMC4359250

[33] Ciardiello C Cavallini L Spinelli C, et al. Focus on extracellular vesicles: new frontiers of cell-to-cell communication in cancer. Int J Mol Sci. 2016;17(2):175. 26861306 10.3390/ijms17020175 PMC4783909

[34] Conigliaro A Costa V Lo Dico A, et al. CD90+ liver cancer cells modulate endothelial cell phenotype through the release of exosomes containing H19 lncRNA. Mol Cancer. 2015;14:155. 26272696 10.1186/s12943-015-0426-x PMC4536801

[35] Console L Scalise M Indiveri C. Exosomes in inflammation and role as biomarkers. Clin Chim Acta. 2019;488:165–171. 30419221 10.1016/j.cca.2018.11.009

[36] Costa-Silva B Aiello NM Ocean AJ, et al. Pancreatic cancer exosomes initiate pre-metastatic niche formation in the liver. Nat Cell Biol. 2015;17(6):816–826. 25985394 10.1038/ncb3169 PMC5769922

[37] Coumans FAW Brisson AR Buzas EI, et al. Methodological guidelines to study extracellular vesicles. Circ Res. 2017;120(10):1632–1648. 28495994 10.1161/CIRCRESAHA.117.309417

[38] Cremer SE Koch J Graversen N, et al. Analytical validation of platelet microparticle quantification in cats. Vet Clin Pathol. 2018;47(3):386–395. 30199121 10.1111/vcp.12641

[39] Crookenden MA Walker CG Peiris H, et al. Effect of circulating exosomes from transition cows on Madin-Darby bovine kidney cell function. J Dairy Sci. 2017;100(7):5687–5700. 28456398 10.3168/jds.2016-12152

[40] Crookenden MA Walker CG Peiris H, et al. Short communication: Proteins from circulating exosomes represent metabolic state in transition dairy cows. J Dairy Sci. 2016;99(9):7661–7668. 27320663 10.3168/jds.2015-10786

[41] Cufaro MC Pieragostino D Lanuti P, et al. Extracellular vesicles and their potential use in monitoring cancer progression and therapy: the contribution of proteomics. J Oncol. 2019;2019:1639854. 31281356 10.1155/2019/1639854 PMC6590542

[42] Darios F Wasser C Shakirzyanova A, et al. Sphingosine facilitates SNARE complex assembly and activates synaptic vesicle exocytosis. Neuron. 2009;62(5):683–694. 19524527 10.1016/j.neuron.2009.04.024 PMC2697323

[43] Desrochers LM Bordeleau F Reinhart-King CA, et al. Microvesicles provide a mechanism for intercellular communication by embryonic stem cells during embryo implantation. Nat Commun. 2016;7(1):1–1. 10.1038/ncomms11958 PMC4912619 27302045

[44] Dhondt B Van Deun J Vermaerke S, et al. Urinary extracellular vesicle biomarkers in urological cancers: from discovery towards clinical implementation. Int J Biochem Cell Biol. 2018;99:236–256. 29654900 10.1016/j.biocel.2018.04.009

[45] Dickens AM Tovar-Y-Romo LB Yoo SW, et al. Astrocyte-shed extracellular vesicles regulate the peripheral leukocyte response to inflammatory brain lesions. Sci Signal. 2017;10(473):eaai7696. 10.1126/scisignal.aai7696 PMC5590230 28377412

[46] Dickhout A Koenen RR. Extracellular vesicles as biomarkers in cardiovascular disease; chances and risks. Front Cardiovasc Med. 2018;5:113. 30186839 10.3389/fcvm.2018.00113 PMC6113364

[47] Dioufa N Clark AM Ma B, et al. Bi-directional exosome-driven intercommunication between the hepatic niche and cancer cells. Mol Cancer. 2017;16(1):172. 29137633 10.1186/s12943-017-0740-6 PMC5686836

[48] Dongre A Weinberg RA. New insights into the mechanisms of epithelial–mesenchymal transition and implications for cancer. Nat Rev Molec Cell Biol. 2019;20(2):69–84. 30459476 10.1038/s41580-018-0080-4

[49] Donnarumma E Fiore D Nappa M, et al. Cancer-associated fibroblasts release exosomal microRNAs that dictate an aggressive phenotype in breast cancer. Oncotarget. 2017;8(12):19592–19608. 28121625 10.18632/oncotarget.14752 PMC5386708

[50] Dror S Sander L Schwartz H, et al. Melanoma miRNA trafficking controls tumour primary niche formation. Nat Cell Biol. 2016;18(9):1006–1017. 27548915 10.1038/ncb3399

[51] Ekström EJ Bergenfelz C von Bülow V, et al. WNT5A induces release of exosomes containing pro-angiogenic and immunosuppressive factors from malignant melanoma cells. Mol Cancer. 2014;13:88. 24766647 10.1186/1476-4598-13-88 PMC4022450

[52] El-Sayed IY Daher A Destouches D, et al. Extracellular vesicles released by mesenchymal-like prostate carcinoma cells modulate EMT state of recipient epithelial-like carcinoma cells through regulation of AR signaling. Cancer Lett. 2017;410:100–111. 28935391 10.1016/j.canlet.2017.09.010

[53] Erdbrügger U Le TH. Extracellular vesicles as a novel diagnostic and research tool for patients with HTN and kidney disease. Am J Physiol Renal Physiol. 2019;317(3):F641–F647. 31313949 10.1152/ajprenal.00071.2019 PMC6766633

[54] Erozenci LA Böttger F Bijnsdorp IV, et al. Urinary exosomal proteins as (pan-)cancer biomarkers: insights from the proteome. FEBS Lett. 2019;593(13):1580–1597. 31198995 10.1002/1873-3468.13487

[55] Fang S Tian H Li X, et al. Clinical application of a microfluidic chip for immunocapture and quantification of circulating exosomes to assist breast cancer diagnosis and molecular classification. PLoS One. 2017;12(4):e0175050. 28369094 10.1371/journal.pone.0175050 PMC5378374

[56] Fauré J Lachenal G Court M, et al. Exosomes are released by cultured cortical neurones. Mol Cell Neurosci. 2006;31(4):642–648. 16446100 10.1016/j.mcn.2005.12.003

[57] Felicetti F De Feo A Coscia C, et al. Exosome-mediated transfer of miR-222 is sufficient to increase tumor malignancy in melanoma. J Transl Med. 2016;14:56. 26912358 10.1186/s12967-016-0811-2 PMC4765208

[58] Finding EJT Lawson C Elliott J, et al. Cell specific microvesicles vary with season and disease predisposition in healthy and previously laminitic ponies. Vet Immunol Immunopathol. 2018;202:85–92. 30078603 10.1016/j.vetimm.2018.06.001

[59] Fish EJ Irizarry KJ DeInnocentes P, et al. Malignant canine mammary epithelial cells shed exosomes containing differentially expressed microRNA that regulate oncogenic networks. BMC Cancer. 2018;18(1):832. 30126376 10.1186/s12885-018-4750-6 PMC6102898

[60] Fortunato O Gasparini P Boeri M, et al. Exo-miRNAs as a new tool for liquid biopsy in lung cancer. Cancers. 2019;11(6):888. 10.3390/cancers11060888 PMC6627875 31242686

[61] French KC Antonyak MA Cerione RA. Extracellular vesicle docking at the cellular port: extracellular vesicle binding and uptake. Semin Cell Dev Biol. 2017;67:48–55. 28104520 10.1016/j.semcdb.2017.01.002 PMC5484727

[62] Frenette G Lessard C Sullivan R. Polyol pathway along the bovine epididymis. Mol Reprod Dev. 2004;69(4):448–456. 15457514 10.1002/mrd.20170

[63] Fröhlich D Kuo WP Frühbeis C, et al. Multifaceted effects of oligodendroglial exosomes on neurons: impact on neuronal firing rate, signal transduction and gene regulation. Philos Trans R Soc B Biol Sci. 2014;369(1652):20130510. 10.1098/rstb.2013.0510 PMC4142031 25135971

[64] Frühbeis C Fröhlich D Kuo WP, et al. Neurotransmitter-triggered transfer of exosomes mediates oligodendrocyte-neuron communication. PLoS Biol. 2013;11(7):e1001604. 23874151 10.1371/journal.pbio.1001604 PMC3706306

[65] Fujita Y Araya J Ito S, et al. Suppression of autophagy by extracellular vesicles promotes myofibroblast differentiation in COPD pathogenesis. J Extracell Vesicles. 2015;4:28388. 26563733 10.3402/jev.v4.28388 PMC4643181

[66] Gangadaran P Hong CM Ahn BC. An update on in vivo imaging of extracellular vesicles as drug delivery vehicles. Front Pharmacol. 2018;9:169. 29541030 10.3389/fphar.2018.00169 PMC5835830

[67] Giesen PLA Rauch U Bohrmann B, et al. Blood-borne tissue factor: another view of thrombosis. Proc Natl Acad Sci U S A. 1999;96(5):2311–2315. 10051638 10.1073/pnas.96.5.2311 PMC26780

[68] Girouard J Frenette G Sullivan R. Comparative proteome and lipid profiles of bovine epididymosomes collected in the intraluminal compartment of the caput and cauda epididymidis. Int J Androl. 2011;34(5 Pt 2):e475–e486. 21875428 10.1111/j.1365-2605.2011.01203.x

[69] Gopal SK Greening DW Zhu HJ, et al. Transformed MDCK cells secrete elevated MMP1 that generates LAMA5 fragments promoting endothelial cell angiogenesis. Sci Rep. 2016;6:28321. 27324842 10.1038/srep28321 PMC4914959

[70] Grange C Tapparo M Collino F, et al. Microvesicles released from human renal cancer stem cells stimulate angiogenesis and formation of lung premetastatic niche. Cancer Res. 2011;71(15):5346–5356. 21670082 10.1158/0008-5472.CAN-11-0241

[71] Greening DW Gopal SK Xu R, et al. Exosomes and their roles in immune regulation and cancer. Semin Cell Dev Biol. 2015;40:72–81. 25724562 10.1016/j.semcdb.2015.02.009

[72] Grein van der SG Defourny KAY Slot EFJ, et al. Intricate relationships between naked viruses and extracellular vesicles in the crosstalk between pathogen and host. Semin Immunopathol. 2018;40(5):491–504. 29789863 10.1007/s00281-018-0678-9 PMC6208671

[73] Gruenberg J Stenmark H. The biogenesis of multivesicular endosomes. Nat Rev Mol Cell Biol. 2004;5(4):317–323. 15071556 10.1038/nrm1360

[74] Halin Bergström S Hägglöf C Thysell E, et al. Extracellular vesicles from metastatic rat prostate tumors prime the normal prostate tissue to facilitate tumor growth. Sci Rep. 2016;6:31805. 27550147 10.1038/srep31805 PMC4994101

[75] Halvaei S Daryani S Eslami SZ, et al. Exosomes in cancer liquid biopsy: a focus on breast cancer. Mol Ther Nucleic Acids. 2018;10:131–141. 29499928 10.1016/j.omtn.2017.11.014 PMC5862028

[76] Han Y Jia L Zheng Y, et al. Salivary exosomes: emerging roles in systemic disease. Int J Biol Sci. 2018;14(6):633–643. 29904278 10.7150/ijbs.25018 PMC6001649

[77] Hanson PI Cashikar A. Multivesicular body morphogenesis. Annu Rev Cell Dev Biol. 2012;28:337–362. 22831642 10.1146/annurev-cellbio-092910-154152

[78] Harrell CR Jovicic N Djonov V, et al. Mesenchymal stem cell-derived exosomes and other extracellular vesicles as new remedies in the therapy of inflammatory diseases. Cells. 2019;8(12):1605. 10.3390/cells8121605 PMC6952783 31835680

[79] Hartjes TA Mytnyk S Jenster GW, et al. Extracellular vesicle quantification and characterization: common methods and emerging approaches. Bioengineering. 2019;6(1):7. 10.3390/bioengineering6010007 PMC6466085 30654439

[80] He WA Calore F Londhe P, et al. Microvesicles containing miRNAs promote muscle cell death in cancer cachexia via TLR7. Proc Natl Acad Sci U S A. 2014;111(12):4525–4529. 24616506 10.1073/pnas.1402714111 PMC3970508

[81] Hornick NI Huan J Doron B, et al. Serum exosome microRNA as a minimally-invasive early biomarker of AML. Sci Rep. 2015;5:11295. 26067326 10.1038/srep11295 PMC4650871

[82] Hoshino A Costa-Silva B Shen TL, et al. Tumour exosome integrins determine organotropic metastasis. Nature. 2015;527(7578):329–335. 26524530 10.1038/nature15756 PMC4788391

[83] Hurwitz SN Sun L Cole KY, et al. An optimized method for enrichment of whole brain-derived extracellular vesicles reveals insight into neurodegenerative processes in a mouse model of Alzheimer’s disease. J Neurosci Methods. 2018;307:210–220. 29894726 10.1016/j.jneumeth.2018.05.022 PMC7052957

[84] Ichii O Ohta H Horino T, et al. Urinary exosome-derived microRNAs reflecting the changes in renal function in cats. Front Vet Sci. 2018;5:289. 30525049 10.3389/fvets.2018.00289 PMC6262179

[85] Ichii O Ohta H Horino T, et al. Urinary exosome-derived microRNAs reflecting the changes of renal function and histopathology in dogs. Sci Rep. 2017;7:40340. 28074869 10.1038/srep40340 PMC5225487

[86] Ingenito F Roscigno G Affnito A, et al. The role of exo-mirnas in cancer: a focus on therapeutic and diagnostic applications. Int J Mol Sci. 2019;20(19):4687. 10.3390/ijms20194687 PMC6801421 31546654

[87] Jang SC Crescitelli R Cvjetkovic A, et al. Mitochondrial protein enriched extracellular vesicles discovered in human melanoma tissues can be detected in patient plasma. J Extracell Vesicles. 2019;8(1):1635420. 31497264 10.1080/20013078.2019.1635420 PMC6719261

[88] Jong de OG Kooijmans SAA Murphy DE, et al. Drug delivery with extracellular vesicles: from imagination to innovation. Acc Chem Res. 2019;52(7):1761–1770. 31181910 10.1021/acs.accounts.9b00109 PMC6639984

[89] Josson S Gururajan M Sung SY, et al. Stromal fibroblast-derived miR-409 promotes epithelial-to-mesenchymal transition and prostate tumorigenesis. Oncogene. 2015;34(21):2690–2699. 25065597 10.1038/onc.2014.212

[90] Kahroba H Hejazi MS Samadi N. Exosomes: from carcinogenesis and metastasis to diagnosis and treatment of gastric cancer. Cell Mol Life Sci. 2019;76(9):1747–1758. 30734835 10.1007/s00018-019-03035-2 PMC11105779

[91] Kapogiannis D Mustapic M Shardell MD, et al. Association of extracellular vesicle biomarkers with Alzheimer disease in the baltimore longitudinal study of aging. JAMA Neurol. 2019;76(11):1340–1351. 10.1001/jamaneurol.2019.2462 PMC6632160 31305918

[92] Khan S Bennit HF Turay D, et al. Early diagnostic value of survivin and its alternative splice variants in breast cancer. BMC Cancer. 2014;14:176. 24620748 10.1186/1471-2407-14-176 PMC3995700

[93] Khan S Simpson J Lynch JC, et al. Racial differences in the expression of inhibitors of apoptosis (IAP) proteins in extracellular vesicles (EV) from prostate cancer patients. PLoS One. 2017;12(10):e0183122. 28981528 10.1371/journal.pone.0183122 PMC5628787

[94] Kidd L Geddings J Hisada Y, et al. Procoagulant microparticles in dogs with immune-mediated hemolytic anemia. J Vet Intern Med. 2015;29(3):908–916. 25871966 10.1111/jvim.12583 PMC4895429

[95] Kim HK Song KS Chung JH, et al. Platelet microparticles induce angiogenesis in vitro. Br J Haematol. 2004;124(3):376–384. 14717787 10.1046/j.1365-2141.2003.04773.x

[96] Kobayashi K Baba K Igase M, et al. Microparticle-associated tissue factor activity in dogs with disseminated intravascular coagulation. J Vet Med Sci. 2020;82(1):56–60. 31787663 10.1292/jvms.19-0553 PMC6983662

[97] Koh YQ Peiris HN Vaswani K, et al. Characterization of exosomes from body fluids of dairy cows. J Anim Sci. 2017;95(9):3893–3904. 28992005 10.2527/jas2017.1727

[98] Kosaka N Yoshioka Y Fujita Y, et al. Versatile roles of extracellular vesicles in cancer. J Clin Invest. 2016;126(4):1163–1172. 26974161 10.1172/JCI81130 PMC4811151

[99] Krämer-Albers EM Bretz N Tenzer S, et al. Oligodendrocytes secrete exosomes containing major myelin and stress-protective proteins: trophic support for axons? Proteomics Clin Appl. 2007;1(11):1446–1461. 21136642 10.1002/prca.200700522

[100] Lachenal G Pernet-Gallay K Chivet M, et al. Release of exosomes from differentiated neurons and its regulation by synaptic glutamatergic activity. Mol Cell Neurosci. 2011;46(2):409–418. 21111824 10.1016/j.mcn.2010.11.004

[101] Lai X Wang M McElyea SD, et al. A microRNA signature in circulating exosomes is superior to exosomal glypican-1 levels for diagnosing pancreatic cancer. Cancer Lett. 2017;393:86–93. 28232049 10.1016/j.canlet.2017.02.019 PMC5386003

[102] Lane RE Korbie D Hill MM, et al. Extracellular vesicles as circulating cancer biomarkers: opportunities and challenges. Clin Transl Med. 2018;7(1):14. 29855735 10.1186/s40169-018-0192-7 PMC5981152

[103] Larssen P Wik L Czarnewski P, et al. Tracing cellular origin of human exosomes using multiplex proximity extension assays. Mol Cell Proteomics. 2017;16(8):1547. 28765260 10.1074/mcp.A116.064725 PMC5546203

[104] Lázaro-Ibáñez E Neuvonen M Takatalo M, et al. Metastatic state of parent cells influences the uptake and functionality of prostate cancer cell-derived extracellular vesicles. J Extracell Vesicles. 2017;6(1):1354645. 28819549 10.1080/20013078.2017.1354645 PMC5556667

[105] Li K Chen Y Li A, et al. Exosomes play roles in sequential processes of tumor metastasis. Int J Cancer. 2019;144(7):1486–1495. 30155891 10.1002/ijc.31774

[106] Li Y Zhao J Yu S, et al. Extracellular vesicles long RNA sequencing reveals abundant mRNA, circRNA, and lncRNA in human blood as potential biomarkers for cancer diagnosis. Clin Chem. 2019;65(6):798–808. 30914410 10.1373/clinchem.2018.301291

[107] Liu Q Yu Z Yuan S, et al. Circulating exosomal microRNAs as prognostic biomarkers for non-small-cell lung cancer. Oncotarget. 2017;8(8):13048–13058. 28055956 10.18632/oncotarget.14369 PMC5355076

[108] Liu S Zhan Y Luo J, et al. Roles of exosomes in the carcinogenesis and clinical therapy of non-small cell lung cancer. Biomed Pharmacother. 2019;111:338–346. 30590322 10.1016/j.biopha.2018.12.088

[109] Liu X Cao M Palomares M, et al. Metastatic breast cancer cells overexpress and secrete miR-218 to regulate type I collagen deposition by osteoblasts. Breast Cancer Res. 2018;20(1):127. 30348200 10.1186/s13058-018-1059-y PMC6198446

[110] Lope de LR Alcíbar OL López AA, et al. Tumour–adipose tissue crosstalk: fuelling tumour metastasis by extracellular vesicles. Philos Trans R Soc B Biol Sci. 2018;373(1737):20160485. 10.1098/rstb.2016.0485 PMC5717439 29158314

[111] Lopez-Verrilli MA Picou F Court FA. Schwann cell-derived exosomes enhance axonal regeneration in the peripheral nervous system. Glia. 2013;61(11):1795–1806. 24038411 10.1002/glia.22558

[112] Lu W Kang Y. Epithelial-mesenchymal plasticity in cancer progression and metastasis. Dev Cell. 2019;49(3):361–374. 31063755 10.1016/j.devcel.2019.04.010 PMC6506183

[113] Ludwig N Whiteside TL Reichert TE. Challenges in exosome isolation and analysis in health and disease. Int J Mol Sci. 2019;20(19):4684. 10.3390/ijms20194684 PMC6801453 31546622

[114] Ma Z Wang Y Li H. Applications of extracellular vesicles in tissue regeneration. Biomicrofluidics. 2020;14(1):011501. 32002105 10.1063/1.5127077 PMC6984977

[115] Markkandan K Ahn K Lee DJ, et al. Profiling and identification of pregnancy-associated circulating microRNAs in dairy cattle. Genes Genomics. 2018;40(10):1111–1117. 30264330 10.1007/s13258-018-0668-2

[116] Martinez VG O’Neill S Salimu J, et al. Resistance to HER2-targeted anti-cancer drugs is associated with immune evasion in cancer cells and their derived extracellular vesicles. Oncoimmunology. 2017;6(12):e1362530. 29209569 10.1080/2162402X.2017.1362530 PMC5706614

[117] Mateescu B Kowal EJK van Balkom BWM, et al. Obstacles and opportunities in the functional analysis of extracellular vesicle RNA - An ISEV position paper. J Extracell Vesicles. 2017;6(1):1286095. 28326170 10.1080/20013078.2017.1286095 PMC5345583

[118] Mathieu M Martin-Jaular L Lavieu G, et al. Specificities of secretion and uptake of exosomes and other extracellular vesicles for cell-to-cell communication. Nat Cell Biol. 2019;21(1):9–17. 30602770 10.1038/s41556-018-0250-9

[119] Maus RLG Jakub JW Hieken TJ, et al. Identification of novel, immune-mediating extracellular vesicles in human lymphatic effluent draining primary cutaneous melanoma. Oncoimmunology. 2019;8(12):e1667742. 31741769 10.1080/2162402X.2019.1667742 PMC6844317

[120] McBride JD Rodriguez-Menocal L Badiavas E V. Extracellular vesicles as biomarkers and therapeutics in dermatology: a focus on exosomes. J Invest Dermatol. 2017;137(8):1622–1629. 28648952 10.1016/j.jid.2017.04.021

[121] McKiernan J Donovan MJ O’Neill V, et al. A novel urine exosome gene expression assay to predict high-grade prostate cancer at initial biopsy. JAMA Oncol. 2016;2(7):882–889. 27032035 10.1001/jamaoncol.2016.0097

[122] McNicholas K Li JYZ Michael MZ, et al. Albuminuria is not associated with elevated urinary vesicle concentration but can confound nanoparticle tracking analysis. 2017;22(11):854–863. 10.1111/nep.12867 27496221

[123] Meckes DG. Exosomal communication goes viral. J Virol. 2015;89(10):5200–5203. 25740980 10.1128/JVI.02470-14 PMC4442506

[124] Meckes DG Gunawardena HP Dekroon RM, et al. Modulation of B-cell exosome proteins by gamma herpesvirus infection. Proc Nati Acad Sci USA. 2013;110(31):E2925–E2933. 10.1073/pnas.1303906110 PMC3732930 23818640

[125] Melo SA Luecke LB Kahlert C, et al. Glypican-1 identifies cancer exosomes and detects early pancreatic cancer. Nature. 2015;523(7559):177–182. 26106858 10.1038/nature14581 PMC4825698

[126] Miki Y Yashiro M Okuno T, et al. Clinico-pathological significance of exosome marker CD63 expression on cancer cells and stromal cells in gastric cancer. PLoS One. 2018;13(9):e0202956. 30222750 10.1371/journal.pone.0202956 PMC6141093

[127] Minciacchi VR Freeman MR Di Vizio D. Extracellular vesicles in cancer: exosomes, microvesicles and the emerging role of large oncosomes. Semin Cell Dev Biol. 2015;40:41–51. 25721812 10.1016/j.semcdb.2015.02.010 PMC4747631

[128] Mittelbrunn M Gutiérrez-Vázquez C Villarroya-Beltri C, et al. Unidirectional transfer of microRNA-loaded exosomes from T cells to antigen-presenting cells. Nat Commun. 2011;2:282. 21505438 10.1038/ncomms1285 PMC3104548

[129] Miyado K Yoshida K Yamagata K, et al. The fusing ability of sperm is bestowed by CD9-containing vesicles released from eggs in mice. Proc Natl Acad Sci U S A. 2008;105(35):12921–1296. 18728192 10.1073/pnas.0710608105 PMC2525563

[130] Mohme M Riethdorf S Pantel K. Circulating and disseminated tumour cells-mechanisms of immune surveillance and escape. Nat Rev Clin Oncol. 2017;14(3):155–167. 27644321 10.1038/nrclinonc.2016.144

[131] Montaner-Tarbes S Novell E Tarancón V, et al. Targeted-pig trial on safety and immunogenicity of serum-derived extracellular vesicles enriched fractions obtained from porcine respiratory and reproductive virus infections. Sci Rep. 2018;8(1):17487. 30504834 10.1038/s41598-018-36141-5 PMC6269534

[132] Montecalvo A Larregina AT Shufesky WJ, et al. Mechanism of transfer of functional microRNAs between mouse dendritic cells via exosomes. Blood. 2012;119(3):756–766. 22031862 10.1182/blood-2011-02-338004 PMC3265200

[133] Monteiro LJ Varas-Godoy M Monckeberg M, et al. Oral extracellular vesicles in early pregnancy can identify patients at risk of developing gestational diabetes mellitus. PLoS One. 2018;14(6):e0218616. 10.1371/journal.pone.0218616 PMC6594608 31242249

[134] Moon PG Lee JE Cho YE, et al. Fibronectin on circulating extracellular vesicles as a liquid biopsy to detect breast cancer. Oncotarget. 2016;7(26):40189–40199. 27250024 10.18632/oncotarget.9561 PMC5130002

[135] Morad G Carman CV Hagedorn EJ, et al. Tumor-derived extracellular vesicles breach the intact blood-brain barrier via transcytosis. ACS Nano. 2019;13(12):13853–13865. 31479239 10.1021/acsnano.9b04397 PMC7169949

[136] Morel L Regan M Higashimori H, et al. Neuronal exosomal mirna-dependent translational regulation of astroglial glutamate transporter glt1. J Biol Chem. 2013;288(10):7105–7116. 23364798 10.1074/jbc.M112.410944 PMC3591620

[137] Mu W Rana S Zöller M. Host matrix modulation by tumor exosomes promotes motility and invasiveness. 2013;15(8):875–887. 10.1593/neo.13786 PMC3730040 23908589

[138] Muntasell A Berger AC Roche PA. T cell-induced secretion of MHC class II-peptide complexes on B cell exosomes. EMBO J. 2007;26(19):4263–4272. 17805347 10.1038/sj.emboj.7601842 PMC2230838

[139] Muralidharan-Chari V Clancy J Plou C, et al. ARF6-regulated shedding of tumor cell-derived plasma membrane microvesicles. Curr Biol. 2009;19(22):1875–1885. 19896381 10.1016/j.cub.2009.09.059 PMC3150487

[140] Nabhan JF Hu R Oh RS, et al. Formation and release of arrestin domain-containing protein 1-mediated microvesicles (ARMMs) at plasma membrane by recruitment of TSG101 protein. Proc Natl Acad Sci U S A. 2012;109(11):4146–4151. 22315426 10.1073/pnas.1200448109 PMC3306724

[141] Nair S Tang KD Kenny L, et al. Salivary exosomes as potential biomarkers in cancer. Oral Oncol. 2018;84:31–40. 30115473 10.1016/j.oraloncology.2018.07.001

[142] Narita M Nishida H Asahina R, et al. Identification of reference genes for microRNAs of extracellular vesicles isolated from plasma samples of healthy dogs by ultracentrifugation, precipitation, and membrane affinity chromatography methods. Am J Vet Res. 2019;80(5):449–454. 31034274 10.2460/ajvr.80.5.449

[143] Neves KB Touyz RM. Extracellular vesicles as biomarkers and biovectors in primary aldosteronism. Hypertension. 2019;74(2):250–252. 31230536 10.1161/HYPERTENSIONAHA.119.13088

[144] Nieuwland R Berckmans RJ Rotteveel-Eijkman RC, et al. Cell-derived microparticles generated in patients during cardiopulmonary bypass are highly procoagulant. Circulation. 1997;96(10):3534–3541. 9396452 10.1161/01.cir.96.10.3534

[145] Nolte-’t Hoen EN van der Vlist EJ Aalberts M, et al. Quantitative and qualitative flow cytometric analysis of nanosized cell-derived membrane vesicles. Nanomedicine. 2012;8(5):712–720. 22024193 10.1016/j.nano.2011.09.006 PMC7106164

[146] Nolte-’t Hoen ENM Buschow SI Anderton SM, et al. Activated T cells recruit exosomes secreted by dendritic cells via LFA-1. Blood. 2009;113(9):1977–1981. 19064723 10.1182/blood-2008-08-174094

[147] Nolte’t Hoen ENM Buermans HPJ Waasdorp M, et al. Deep sequencing of RNA from immune cell-derived vesicles uncovers the selective incorporation of small non-coding RNA biotypes with potential regulatory functions. Nucleic Acids Res. 2012;40(18):9272–9285. 22821563 10.1093/nar/gks658 PMC3467056

[148] Ogata-Kawata H Izumiya M Kurioka D, et al. Circulating exosomal microRNAs as biomarkers of colon cancer. PLoS One. 2014;9(4):e92921. 24705249 10.1371/journal.pone.0092921 PMC3976275

[149] Okoye IS Coomes SM Pelly VS, et al. MicroRNA-containing T-regulatory-cell-derived exosomes suppress pathogenic T helper 1 cells. Immunity. 2014;41(1):89–103. 25035954 10.1016/j.immuni.2014.05.019 PMC4104030

[150] O’Loghlen A. Role for extracellular vesicles in the tumour microenvironment. Philos Trans R Soc B Biol Sci. 2018;373(1737):20170066. 10.1098/rstb.2016.0488 PMC5717441 29158316

[151] Ono M Kosaka N Tominaga N, et al. Exosomes from bone marrow mesenchymal stem cells contain a microRNA that promotes dormancy in metastatic breast cancer cells. Sci Signal. 2014;7(332):ra63. 10.1126/scisignal.2005231 24985346

[152] Ozawa PMM Alkhilaiwi F Cavalli IJ, et al. Extracellular vesicles from triple-negative breast cancer cells promote proliferation and drug resistance in non-tumorigenic breast cells. Breast Cancer Res Treat. 2018;172(3):713–723. 30173296 10.1007/s10549-018-4925-5 PMC6245099

[153] Palacios A Sampedro L Sevilla IA, et al. Mycobacterium tuberculosis extracellular vesicle-associated lipoprotein LpqH as a potential biomarker to distinguish paratuberculosis infection or vaccination from tuberculosis infection. BMC Vet Res. 2019;15(1):188. 31174546 10.1186/s12917-019-1941-6 PMC6555097

[154] Panagopoulou MS Wark AW Birch DJS, et al. Phenotypic analysis of extracellular vesicles: a review on the applications of fluorescence. J Extracell Vesicles. 2020;9(1):1710020. 32002172 10.1080/20013078.2019.1710020 PMC6968689

[155] Pavlyukov MS Yu H Bastola S, et al. Apoptotic cell-derived extracellular vesicles promote malignancy of glioblastoma via intercellular transfer of splicing factors. Cancer Cell. 2018;34(1):119–135.e10. 29937354 10.1016/j.ccell.2018.05.012 PMC6048596

[156] Pei H Jin Z Chen S, et al. MiR-135b promotes proliferation and invasion of osteosarcoma cells via targeting FOXO1. Mol Cell Biochem. 2014;400(1–2):245–252. 25416447 10.1007/s11010-014-2281-2

[157] Peinado H Alečković M Lavotshkin S, et al. Melanoma exosomes educate bone marrow progenitor cells toward a pro-metastatic phenotype through MET. Nat Med. 2012;18(6):883–891. 22635005 10.1038/nm.2753 PMC3645291

[158] Peinado H Zhang H Matei IR, et al. Pre-metastatic niches: organ-specific homes for metastases. Nat Rev Cancer. 2017;17(5):302–317. 28303905 10.1038/nrc.2017.6

[159] Phinney DG Di Giuseppe M Njah J, et al. Mesenchymal stem cells use extracellular vesicles to outsource mitophagy and shuttle microRNAs. Nat Commun. 2015;6:8472. 26442449 10.1038/ncomms9472 PMC4598952

[160] Pohler KG Green JA Moley LA, et al. Circulating microRNA as candidates for early embryonic viability in cattle. Mol Reprod Dev. 2017;84(8):731–743. 28643872 10.1002/mrd.22856 PMC5580359

[161] Potz BA Scrimgeour LA Pavlov VI, et al. Extracellular vesicle injection improves myocardial function and increases angiogenesis in a swine model of chronic ischemia. J Am Heart Assoc. 2018;7(12):e008344. 29895586 10.1161/JAHA.117.008344 PMC6220556

[162] Probert C Dottorini T Speakman A, et al. Communication of prostate cancer cells with bone cells via extracellular vesicle RNA; a potential mechanism of metastasis. Oncogene. 2019;38(10):1751–1763. 30353168 10.1038/s41388-018-0540-5 PMC6372071

[163] Pucci F Garris C Lai CP, et al. SCS macrophages suppress melanoma by restricting tumor-derived vesicle-B cell interactions. Science (80-). 2016;352(6282):242–246. 10.1126/science.aaf1328 PMC4960636 26989197

[164] Raposo G Nijman HW Stoorvogel W, et al. B lymphocytes secrete antigen-presenting vesicles. J Exp Med. 1996;183(3):1161–1172. 8642258 10.1084/jem.183.3.1161 PMC2192324

[165] Ratajczak J Miekus K Kucia M, et al. Embryonic stem cell-derived microvesicles reprogram hematopoietic progenitors: evidence for horizontal transfer of mRNA and protein delivery. Leukemia. 2006;20(5):847–856. 16453000 10.1038/sj.leu.2404132

[166] Read J Ingram A Al Saleh HA, et al. Nuclear transportation of exogenous epidermal growth factor receptor and androgen receptor via extracellular vesicles. Eur J Cancer. 2017;70:62–74. 27886573 10.1016/j.ejca.2016.10.017

[167] Robbins PD Morelli AE. Regulation of immune responses by extracellular vesicles. Nat Rev Immunol. 2014;14(3):195–208. 24566916 10.1038/nri3622 PMC4350779

[168] Roura S Gámez-Valero A Lupón J, et al. Proteomic signature of circulating extracellular vesicles in dilated cardiomyopathy. Lab Investig. 2018;98(10):1291–1299. 29540862 10.1038/s41374-018-0044-5

[169] Rout ED Webb TL Laurence HM, et al. Transferrin receptor expression in serum exosomes as a marker of regenerative anaemia in the horse. Equine Vet J. 2015;47(1):101–106. 24708277 10.1111/evj.12235

[170] Samanta S Rajasingh S Drosos N, et al. Exosomes: new molecular targets of diseases. Acta Pharmacol Sin. 2018;39(4):501–513. 29219950 10.1038/aps.2017.162 PMC5888687

[171] Sammarco A Finesso G Cavicchioli L, et al. Preliminary investigation of extracellular vesicles in mammary cancer of dogs and cats: identification and characterization. Vet Comp Oncol. 2018;16(4):489–496. 29851284 10.1111/vco.12405

[172] Samuel M Chisanga D Liem M, et al. Bovine milk-derived exosomes from colostrum are enriched with proteins implicated in immune response and growth. Sci Rep. 2017;7(1):5933. 28725021 10.1038/s41598-017-06288-8 PMC5517456

[173] Samuel M Gabrielsson S. Personalized medicine and back–allogeneic exosomes for cancer immunotherapy. J Int Med. 2019;289(2):138–146. 10.1111/joim.12963 31359504

[174] Sánchez CA Andahur EI Valenzuela R, et al. Exosomes from bulk and stem cells from human prostate cancer have a differential microRNA content that contributes cooperatively over local and pre-metastatic niche. Oncotarget. 2016;7(4):3993–4008. 26675257 10.18632/oncotarget.6540 PMC4826185

[175] Sansone P Berishaj M Rajasekhar VK, et al. Evolution of cancer stem-like cells in endocrine-resistant metastatic breast cancers is mediated by stromal microvesicles. Cancer Res. 2017;77(8):1927–1941. 28202520 10.1158/0008-5472.CAN-16-2129 PMC5392366

[176] Santos JC Lima NDS Sarian LO, et al. Exosome-mediated breast cancer chemoresistance via miR-155 transfer. Sci Rep. 2018;8(1):829. 29339789 10.1038/s41598-018-19339-5 PMC5770414

[177] Scarlotta M Simsek C Kim AK. Liquid biopsy in solid malignancy. Genet Test Mol Biomarkers. 2019;23(4):284–296. 30916594 10.1089/gtmb.2018.0237 PMC10162121

[178] Shao C Yang F Miao S, et al. Role of hypoxia-induced exosomes in tumor biology. Mol Cancer. 2018;17(1):120. 30098600 10.1186/s12943-018-0869-y PMC6087002

[179] Shi R Wang PY Li XY, et al. Exosomal levels of miRNA-21 from cerebrospinal fluids associated with poor prognosis and tumor recurrence of glioma patients. Oncotarget. 2015;6(29):26971–26981. 26284486 10.18632/oncotarget.4699 PMC4694967

[180] Shimoda A Ueda K Nishiumi S, et al. Exosomes as nanocarriers for systemic delivery of the *Helicobacter pylori* virulence factor CagA. Sci Rep. 2016;6:18346. 26739388 10.1038/srep18346 PMC4703974

[181] Shimoda M. Extracellular vesicle-associated MMPs: a modulator of the tissue microenvironment. Adv Clin Chem. 2019;88:35–66. 30612606 10.1016/bs.acc.2018.10.006

[182] Sil S Dagur RS Liao K, et al. Strategies for the use of extracellular vesicles for the delivery of therapeutics. J Neuroimmune Pharmacol. 2020;15(3):422–442. 31456107 10.1007/s11481-019-09873-y PMC7044028

[183] Šimundić M Švara T Štukelj R, et al. Concentration of extracellular vesicles isolated from blood relative to the clinical pathological status of dogs with mast cell tumours. Vet Comp Oncol. 2019;17(4):456–464. 31066969 10.1111/vco.12489

[184] Song YH Warncke C Choi SJ, et al. Breast cancer-derived extracellular vesicles stimulate myofibroblast differentiation and pro-angiogenic behavior of adipose stem cells. Matrix Biol. 2017;60–61:190–205. 10.1016/j.matbio.2016.11.008 PMC5438891 27913195

[185] Stoner SA Duggan E Condello D, et al. High sensitivity flow cytometry of membrane vesicles. Cytom Part A. 2016;89(2):196–206. 10.1002/cyto.a.22787 26484737

[186] Sun J Aswath K Schroeder SG, et al. MicroRNA expression profiles of bovine milk exosomes in response to Staphylococcus aureus infection. BMC Genomics. 2015;16:806. 26475455 10.1186/s12864-015-2044-9 PMC4609085

[187] Sun Z Shi K Yang S, et al. Effect of exosomal miRNA on cancer biology and clinical applications. Mol Cancer. 2018;17(1):147. 30309355 10.1186/s12943-018-0897-7 PMC6182840

[188] Szatanek R Baj-Krzyworzeka M Zimoch J, et al. The methods of choice for extracellular vesicles (EVs) characterization. Int J Mol Sci. 2017;18(6):1153. 10.3390/ijms18061153 PMC5485977 28555055

[189] Takasugi M Okada R Takahashi A, et al. Small extracellular vesicles secreted from senescent cells promote cancer cell proliferation through EphA2. Nat Commun. 2017;8:15729. 10.1038/ncomms15728 PMC5467215 28585531

[190] Taraboletti G D’Ascenzo S Borsotti P, et al. Shedding of the matrix metalloproteinases MMP-2, MMP-9, and MT1-MMP as membrane vesicle-associated components by endothelial cells. Am J Pathol. 2002;160(2):673–680. 11839588 10.1016/S0002-9440(10)64887-0 PMC1850663

[191] Théry C Witwer KW Aikawa E, et al. Minimal information for studies of extracellular vesicles 2018 (MISEV2018): a position statement of the international society for extracellular vesicles and update of the MISEV2014 guidelines. J Extracell Vesicles. 2018;7(1):1535750. 30637094 10.1080/20013078.2018.1535750 PMC6322352

[192] Thimon V Frenette G Saez F, et al. Protein composition of human epididymosomes collected during surgical vasectomy reversal: a proteomic and genomic approach. Hum Reprod. 2008;23(8):1698–1707. 18482993 10.1093/humrep/den181

[193] Troyer RM Ruby CE Goodall CP, et al. Exosomes from osteosarcoma and normal osteoblast differ in proteomic cargo and immunomodulatory effects on T cells. Exp Cell Res. 2017;358(2):369–376. 28712929 10.1016/j.yexcr.2017.07.011

[194] Vagner T Spinelli C Minciacchi VR, et al. Large extracellular vesicles carry most of the tumour DNA circulating in prostate cancer patient plasma. J Extracell Vesicles. 2018;7(1):1505403. 30108686 10.1080/20013078.2018.1505403 PMC6084494

[195] Valenzuela MMA Ferguson Bennit HR Gonda A, et al. Exosomes secreted from human cancer cell lines contain inhibitors of apoptosis (IAP). Cancer Microenviron. 2015;8(2):65–73. 25982218 10.1007/s12307-015-0167-9 PMC4542824

[196] Vaswani K Koh YQ Almughlliq FB, et al. A method for the isolation and enrichment of purified bovine milk exosomes. Reprod Biol. 2017;17(4):341–348. 29030127 10.1016/j.repbio.2017.09.007

[197] Veld SGJG Wurdinger T. Tumor-educated platelets. Blood. 2019;133(22):2359–2364. 30833413 10.1182/blood-2018-12-852830

[198] Vella LJ Scicluna BJ Cheng L, et al. A rigorous method to enrich for exosomes from brain tissue. J Extracell Vesicles. 2017;6(1):1348885. 28804598 10.1080/20013078.2017.1348885 PMC5533148

[199] Villatoro AJ Alcoholado C Martín-Astorga MC, et al. Comparative analysis and characterization of soluble factors and exosomes from cultured adipose tissue and bone marrow mesenchymal stem cells in canine species. Vet Immunol Immunopathol. 2019;208:6–15. 30712794 10.1016/j.vetimm.2018.12.003

[200] Vlist van der EJ Nolte-’t Hoen ENM Stoorvogel W, et al. Fluorescent labeling of nano-sized vesicles released by cells and subsequent quantitative and qualitative analysis by high-resolution flow cytometry. Nat Protoc. 2012;7(7):1311–1326. 22722367 10.1038/nprot.2012.065

[201] Wang H Jiang D Li W, et al. Evaluation of serum extracellular vesicles as noninvasive diagnostic markers of glioma. Theranostics. 2019;9(18):5347–5358. 31410219 10.7150/thno.33114 PMC6691576

[202] Wang S Kojima K Mobley JA, et al. Proteomic analysis of urinary extracellular vesicles reveal biomarkers for neurologic disease. EbioMedicine. 2019;45:351–361. 31229437 10.1016/j.ebiom.2019.06.021 PMC6642358

[203] Wang T Gilkes DM Takano N, et al. Hypoxia-inducible factors and RAB22A mediate formation of microvesicles that stimulate breast cancer invasion and metastasis. Proc Natl Acad Sci U S A. 2014;111(31):E3234–E3242. 24938788 10.1073/pnas.1410041111 PMC4128139

[204] Webber JP Spary LK Sanders AJ, et al. Differentiation of tumour-promoting stromal myofibroblasts by cancer exosomes. Oncogene. 2015;34(3):290–302. 24441045 10.1038/onc.2013.560

[205] Whiteside TL. The potential of tumor-derived exosomes for noninvasive cancer monitoring: an update. Expert Rev Mol Diagn. 2018;18(12):1029–1040. 30406709 10.1080/14737159.2018.1544494 PMC6506389

[206] Wu X Liu Y Wei W, et al. Extracellular vesicles in autoimmune vasculitis: little dirts light the fire in blood vessels. Autoimmun Rev. 2019;18(6):593–606. 30959208 10.1016/j.autrev.2018.12.007 PMC6956404

[207] Xiao D Barry S Kmetz D, et al. Melanoma cell-derived exosomes promote epithelial-mesenchymal transition in primary melanocytes through paracrine/autocrine signaling in the tumor microenvironment. Cancer Lett. 2016;376(2):318–327. 27063098 10.1016/j.canlet.2016.03.050 PMC4869527

[208] Xu R Rai A Chen M, et al. Extracellular vesicles in cancer—implications for future improvements in cancer care. Nat Rev Clin Oncol. 2018;15(10):617–638. 29795272 10.1038/s41571-018-0036-9

[209] Xu Y Luo F Liu Y, et al. Exosomal miR-21 derived from arsenite-transformed human bronchial epithelial cells promotes cell proliferation associated with arsenite carcinogenesis. Arch Toxicol. 2015;89(7):1071–1082. 24912785 10.1007/s00204-014-1291-x

[210] Yamada T Shigemura H Ishiguro N, et al. Cell infectivity in relation to bovine leukemia virus gp51 and p24 in bovine milk exosomes. PLoS One. 2013;8(10):e77359. 24146982 10.1371/journal.pone.0077359 PMC3798320

[211] Yamauchi M Shimizu K Rahman M, et al. Efficient method for isolation of exosomes from raw bovine milk. Drug Dev Ind Pharm. 2019;45(3):359–364. 30366501 10.1080/03639045.2018.1539743

[212] Yang VK Loughran KA Meola DM, et al. Circulating exosome microRNA associated with heart failure secondary to myxomatous mitral valve disease in a naturally occurring canine model. J Extracell Vesicles. 2017;6(1):1350088. 28804599 10.1080/20013078.2017.1350088 PMC5533140

[213] Yokoyama S Takeuchi A Yamaguchi S, et al. Clinical implications of carcinoembryonic antigen distribution in serum exosomal fraction—measurement by ELISA. PLoS One. 2017;12(8):e0183337. 28817685 10.1371/journal.pone.0183337 PMC5560664

[214] You Y Ikezu T. Emerging roles of extracellular vesicles in neurodegenerative disorders. Neurobiol Dis. 2019;130:104512. 31229685 10.1016/j.nbd.2019.104512 PMC6689424

[215] Yuana Y Sturk A Nieuwland R. Extracellular vesicles in physiological and pathological conditions. Blood Rev. 2013;27(1):31–39. 23261067 10.1016/j.blre.2012.12.002

[216] Zeng Z Li Y Pan Y, et al. Cancer-derived exosomal miR-25-3p promotes pre-metastatic niche formation by inducing vascular permeability and angiogenesis. Nat Commun. 2018;9(1):5395. 30568162 10.1038/s41467-018-07810-w PMC6300604

[217] Zhao K Liang G Sun X, et al. Comparative miRNAome analysis revealed different miRNA expression profiles in bovine sera and exosomes. BMC Genomics. 2016;17(1):630. 27519500 10.1186/s12864-016-2962-1 PMC4983018

[218] Zhao Z Fan J Hsu YMS, et al. Extracellular vesicles as cancer liquid biopsies: from discovery, validation, to clinical application. Lab on a Chip. 2019;19(7):1114–1140. 30882822 10.1039/c8lc01123k PMC6469512

[219] Zitvogel L Regnault A Lozier A, et al. Eradication of established murine tumors using a novel cell-free vaccine: dendritic cell-derived exosomes. Nat Med. 1998;4(5):594–600. 9585234 10.1038/nm0598-594

[220] Żmigrodzka M Guzera M Miśkiewicz A, et al. The biology of extracellular vesicles with focus on platelet microparticles and their role in cancer development and progression. Tumor Biol. 2016;37(11):14391–14401. 10.1007/s13277-016-5358-6 PMC5126185 27629289

[221] Żmigrodzka M Witkowska-Piłaszewicz O Rzepecka A, et al. Extracellular vesicles in the blood of dogs with cancer—a preliminary study. Animals (Basel). 2019;9(8):575. 10.3390/ani9080575 PMC6720862 31430895

[222] Zonneveld MI Brisson AR van Herwijnen MJC, et al. Recovery of extracellular vesicles from human breast milk is influenced by sample collection and vesicle isolation procedures. J Extracell Vesicles. 2014;3. 10.3402/jev.v3.24215 PMC4139932 25206958

